# The use of co-design in developing physical activity interventions for older adults: a scoping review

**DOI:** 10.1186/s12877-022-03345-4

**Published:** 2022-08-08

**Authors:** Natalie Constantin, Holly Edward, Hayley Ng, Anna Radisic, Amy Yule, Alina D’Asti, Cassandra D’Amore, Julie C. Reid, Marla Beauchamp

**Affiliations:** School of Rehabilitation Science, McMaster University, Hamilton, ON Canada

**Keywords:** Aging, Exercise, Health promotion, Participation, Participatory research, Seniors, Co-design

## Abstract

**Background:**

Promoting physical activity (PA) participation in older adults is important for preserving quality of life and functional independence. Co-design has been shown to increase engagement of end-users in health-related policies and interventions. This scoping review aimed to examine how co-design has been used to develop PA interventions for older adults.

**Methods:**

We searched MEDLINE, EMBASE, AMED, and CINAHL. Peer-reviewed primary research studies that met the following criteria were included: had at least one participant aged ≥60 years involved in the co-design process and the intervention was delivered to individuals whose mean age was ≥60, used co-design methodologies, and any form of PA. After duplicate removal, two or more independent reviewers completed title and abstract and full text screening. Data were extracted from the included studies according to study aims.

**Results:**

Of the 29 included studies, 12 different terms were used to describe co-design with variable operational definitions that we consolidated into five proposed components. Fifteen studies engaged users in a consultative way, 13 studies using collaboration, and one study engaged end-users in consumer-control. No studies involved end-users in the dissemination phase. Further, no studies directly measured the effectiveness of the co-design process. Five categories of barriers and facilitators to co-design were identified including frameworks and methodologies, logistics, relationships, participation, and generalizability.

**Conclusions:**

There is a large degree of variability in how co-design is used to develop PA interventions for older adults. Our findings can be used by researchers to improve rigor and standardization in this emerging field.

**Trial registration:**

osf.io/vsw2m.

**Supplementary Information:**

The online version contains supplementary material available at 10.1186/s12877-022-03345-4.

## Background

Co-design is an emerging methodology within healthcare research [1, 2]. It aims to actively engage specific groups of individuals, such as end-users, to aid in the development of products or services through knowledge sharing [3]. While patient centered care emphasizes that patient values guide clinical decision making, co-design is a methodology that can formally incorporate the ideas and values of end-users into the development of services, policies, and interventions [4]. Co-designed initiatives promote patient-centered care by incorporating varying degrees of stakeholder input into the development or reform of health services while providing quality assurance [5]. These initiatives are embodied in the mandates of organizations such as the Patient Centred Outcomes Research Institute (PCORI) in the United States, which advocates for clinician, patient, and end-user involvement throughout healthcare related research [4].

Physical activity (PA) participation across the lifespan is essential for maintaining functional independence and preventing chronic disease later in life [6–13]. In this review, we define PA as any form of activity that results in the expenditure of energy [14]. The World Health Organization (WHO) recommends that adults aged ≥65 years complete 150–300 minutes of moderate-intensity aerobic activity or 75–150 minutes of vigorous-intensity aerobic activity per week in addition to two or more days of strength and balance training [15]. Older adults who participate in regular PA are less likely to develop conditions such as cardiovascular disease, diabetes, and stroke [16]. Furthermore, they are more likely to notice positive improvements in their mental health and quality of life [16]. However, despite the known importance of PA, the WHO estimates that 25% of adults globally do not meet the minimum recommended guidelines [17]. Additionally, the WHO estimates approximately 3.2 million deaths per year are due to physical inactivity [17].

At the onset of the COVID-19 pandemic, leaders implemented stringent physical distancing measures to prevent the spread of the virus and protect vulnerable populations. While this has helped curb the spread of the virus in some communities, it has contributed to an increase in sedentary behaviour [18, 19]. International research shows that the community lockdowns and physical distancing regulations due to the COVID-19 have drastically decreased physical activity levels in all age groups, including older adults [18–23].

Evaluations of the impact of patient and public involvement in research show that patients, communities, and researchers all benefit from co-design [24]. A preliminary search focused on co-design methodologies and PA interventions in older adults identified one systematic review and one narrative literature review examining barriers and facilitators to end-user involvement [25, 26]. Co-design is gaining popularity in health research and policy development, but a clear description of terminology, methodology, and evaluation tools is lacking [2]. This scoping review maps the current state of the literature on the use of co-design for developing PA interventions for older adults and identifies gaps for future research. To our knowledge, this is the first review to summarize terminology and definitions that describe co-design, as well as map how and when end-users are involved throughout the process.

### Objectives

The overall objective of this scoping review was to examine how co-design has been used to develop PA interventions for older adults. The specific aims of this review were to: 1) report the terminology and definitions that have been used to describe co-design in included studies, 2) describe what phases of the research process co-design has been used, 3) determine the levels of involvement of the end-users, 4) understand how the success and/or effectiveness of co-design has been measured, and 5) identify barriers and facilitators for the co-design process.

## Methods

Full details of the study methodology are outlined in the study protocol published elsewhere [27]. We followed standardized frameworks from Arksey and O’Malley, Levac et al., and the Joanna Briggs Institute when conducting this scoping review [28–30]. We reported our work according to the Preferred Reporting Items for Systematic Reviews (PRISMA-ScR) Extension for scoping reviews (Additional file 1) [31]. Our search strategy was developed in consultation with two health science research librarians [32]. We searched 4 electronic databases from inception through Feb. 18, 2021, including MEDLINE, EMBASE, AMED, and CINAHL. The full search strategy can be found in Additional file 2. Relevant reviews were retained for hand searching of reference lists. We conducted the review between February and June of 2021. Five reviewers participated in all stages of screening (i.e., titles, abstracts, and full texts) and data extraction (AR, AD, NC, HE, HN). Each title was screened by two independent reviewers at each stage. Disagreements at any stage were resolved by consensus, or by a third reviewer (AY) where necessary. Agreement between reviewers was calculated at the title and abstract stage [33].

### Inclusion and exclusion

Criteria were aligned with the participant, concept, and context framework. We included: *Participant –* at least one participant aged ≥60 years involved in co-design of an intervention for a target population whose mean age was ≥60 years; *Concept* – co-design of a PA related intervention as defined in our introduction; *Context* – any clinical population or setting. We excluded studies that did not use co-design methodologies and studies that did not involve PA interventions. We also excluded grey literature, literature reviews, and non-English studies. We imported all citations into Covidence v.2576 c3a8578b (Veritas Health Innovation, Melbourne, Australia). The results of the search and the study inclusion process were reported in full in a PRISMA-ScR flow diagram in Additional file 3 [31].

### Data extraction and synthesis

Co-design terminology and operational definitions were extracted verbatim from text. Operational definitions were analyzed, and repetitive concepts identified. Similar concepts were highlighted with the same colour. For example, concepts such “partnership”, “collaboration”, “working with” and “shared leadership” were grouped together under the “collaboration between researchers, older adults, and other relevant stakeholders” component. Studies were categorized according to the planning, conducting, and dissemination phases of involvement described by the Patient-Centered Outcomes Research Institute (PCORI) [34] and similarly by the consultation, collaboration, and consumer control levels of involvement outlined by Boote, Telford, and Cooper [35]. Outcome measures used to examine the success (the accomplishment of research aims) and/or effectiveness (the extent of producing the desired result) of the co-design process were recorded. Authors were contacted by email when missing or additional information was required.

## Results

### Study inclusion

From four electronic databases, we identified 10,956 citations. An additional 8 citations were retrieved from manual searching of reference lists. We identified 29 papers that met our inclusion criteria. Reasons for exclusion are reported in our PRISMA flow chart (Additional file 3). Primary reasons for exclusion included not focusing on older adults, no co-design, and no intervention design. A detailed list of all citations excluded on full-text examination and reasons for exclusion can be found in Additional file 4. Agreement between reviewers for title and abstract screening was between 0.84 and 1.

### Characteristics of included studies

Included studies were published between 2000 and 2021, with 82% of the studies published since 2016. Multiple countries of origin were represented across included studies such as the United Kingdom (17%), United States (14%), and the Netherlands (10%). Most studies (90%) were set in the community, followed by long-term care or retirement homes (7%), and then hospital (3%). In terms of study design, 76% were mixed methods, 10% randomized controlled trials, 7% protocols, 3% case study and participatory design, and 3% cross-sectional design. Additional file 5 summarizes the characteristics of our included studies.

### Terminology and operational definitions of co-design

**Fig. 1:**
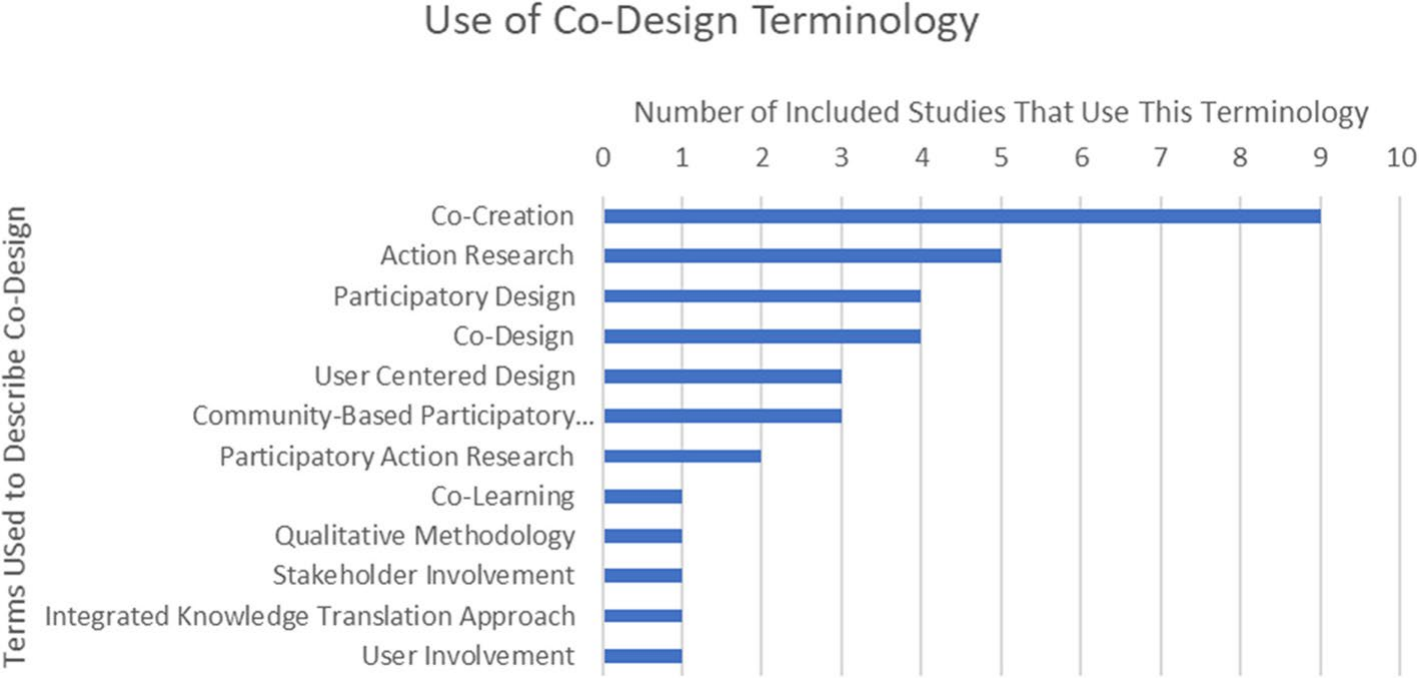
Use of co-design terminology in included studies

We identified 12 different terms to describe the process of co-design. Figure 1 shows the frequency of each term. Twenty-six (90%) studies provided operational definitions (Additional file 6), and 3 studies did not. Thematic analysis of the 26 operational definitions revealed repetitive concepts that were categorized into 10 themes that were further grouped into 5 proposed components of co-design as shown in Table 1. The terminology column represents the various terms used across studies under the umbrella of co-design. In each row, we identify which studies incorporated the components of our proposed co-design definition. From this analysis, our proposed standardized definition for co-design is a user-centered approach involving collaboration between researchers, end-users, and other relevant stakeholders who are actively engaged throughout a process of iteration and continuous reflection to create an intervention tailored to the specific needs of the target population.

**Table 1:** Thematic analysis of co-design operational definitions

Terminology	Proposed Components of Co-Design				
	User-centered approach [36–49]	Collaboration between researchers, older adults, and other relevant stakeholders [37, 39, 40, 42–44, 46, 48–59]	Tailored to specific needs [36, 37, 44, 45, 48, 51, 53, 55, 56, 60, 61]	Active involvement throughout [37, 41, 43, 46–48, 56–59, 62]	Iteration and continuous reflection [36, 47, 48, 54, 56]
Co-Design [37, 38, 48, 63]	[37, 38, 48]	[37, 48]	[37, 48]	[37, 48]	[48]
Co-Creation [13, 28, 30–33, 37, 39, 42, 43, 54]	[37, 39–43, 47, 49]	[37, 39, 40, 42, 43, 49, 52, 53]	[37, 53]	[37, 41, 43, 47]	[47]
Action Research [39, 40, 49, 56, 62, 64, 65]		[39, 40, 49, 56]	[56]	[56, 62]	[56]
Participatory Design [36, 43, 46, 55]	[36, 43, 46]	[43, 46, 55]	[36, 55]	[43, 46]	
Community-Based Participatory Research/Participatory Research [44, 57, 58, 61]	[44]	[44]	[44, 57, 58]	[57, 58]	
User-Centered Design [36, 45, 47]	[36, 45, 47]		[36, 45]		[36, 47]
Participatory Action Research [51, 54]		[51, 54]	[51]		[54]
Integrated Knowledge Translation [50]		[50]			
Qualitative Methodology [60]			[60]		
User-involvement [41]	[41]			[41]	
Co-learning [62]				[62]	
Stakeholder involvement [59]				[59]	
Total	12	17	11	10	5

We systematically identified whether the proposed components of our co-design operational definition were carried out in each study’s methodology. Twelve studies included a user-centered approach, 17 demonstrated collaboration between researchers, older adults, and other relevant stakeholders, 11 tailored their interventions to the specific needs of the target population, 10 actively involved end-users throughout the study, and 5 demonstrated iteration or continuous reflection.

### Use of co-Design in the Phases of research

**Fig. 2:**
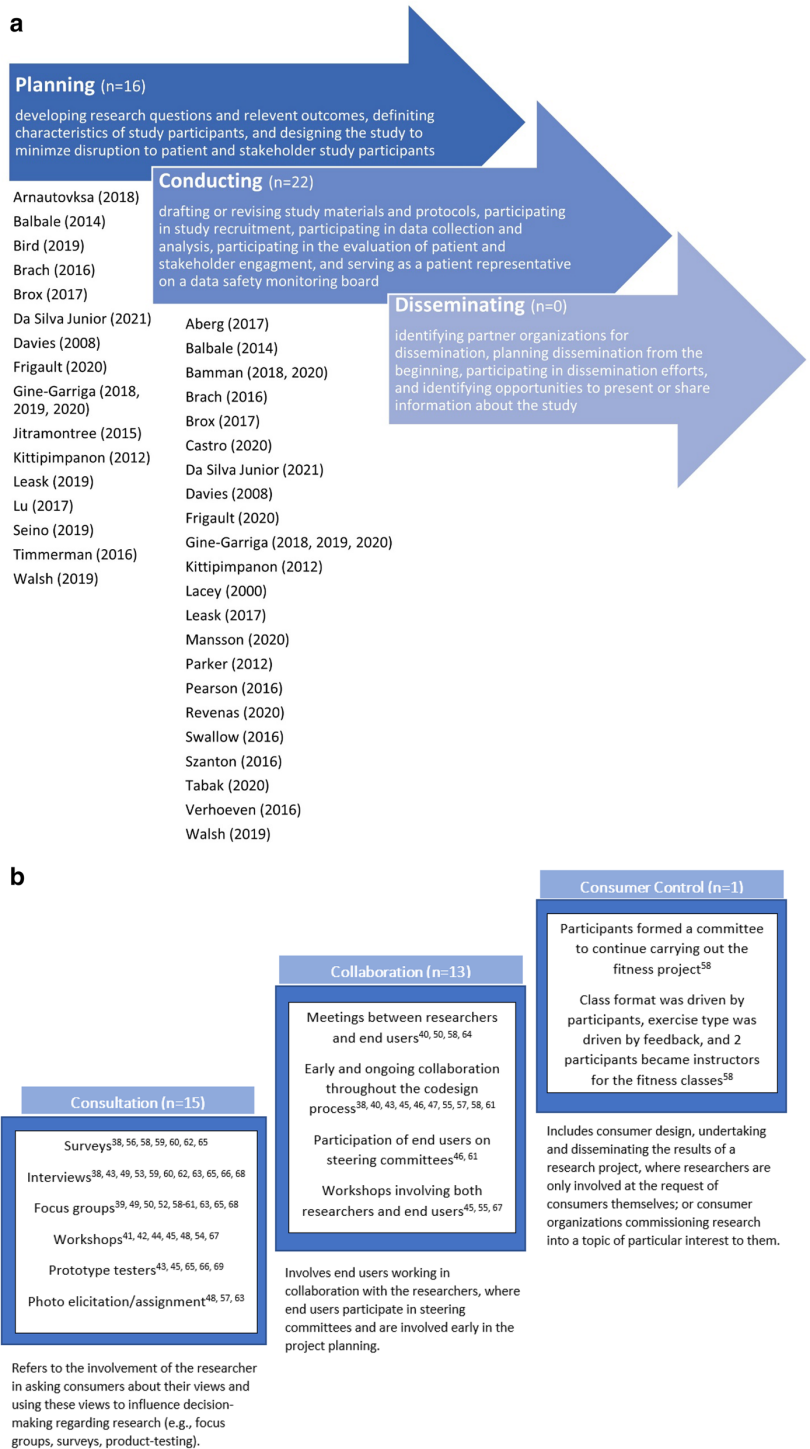
**a** Distribution of studies according to phase of research in which end-users were involved. **b** Levels of involvement of end-users in the co-design process

Figure 2a shows the distribution of studies according to the phase of research in which end-users were involved. Nine studies utilized co-design in more than one phase, such as both planning and conducting.

### Determine the levels of involvement of the end-users in the included studies

The levels of involvement of end-users progress from consultation to collaboration to consumer control the highest level involvement. Most end-users were involved at either the consultation (52%) or collaboration (45%) level in the included studies. Figure 2b summarizes the different levels of involvement, the number of studies within each level, and the methods used within each level.

### Measuring success and/or effectiveness of co-design

None of the included studies described any type of process to evaluate the success and/or effectiveness of the co-design process itself. However, some studies evaluated the success and/or effectiveness of the co-design process through indirect methods, such as measuring participant satisfaction of the PA intervention itself (*n* = 4), intervention adherence measures (*n* = 6), and by assessing changes in PA performance and levels of PA (*n* = 5) (Table 2). Of the 15 studies that indirectly evaluated co-design, all reported positive trial outcomes that they attribute to the use of a co-design process.

**Table 2:** Outcome measures used to examine the success and/or effectiveness of co-design

Indirect Methods	
Participant Satisfaction	Adherence
-Gaming Experience Questionnaire [38] -Fall Prevention Program Satisfaction Questionnaire [64] -Program and Engagement Satisfaction Surveys [52, 59]	-Physical Activity Adherence Questionnaire [38] -Attendance recorded [52, 56, 59] -Recorded the number of days or time the product was used [66, 67]
**PA Measures**	
-Functional Capacity measured by The Senior Fitness Test [38] -Fall Prevention Behaviors Questionnaire to assess five areas: 1) fall prevention practices, 2) regular vision assessment, 3) medication use, 4) exercise and 5) home environment [64] -Physical Performance Test (PPT) to assess upper body muscle strength, lower body muscle strength, balance, and balance and gait [64] -Recorded total steps/day [66] -Amount of PA via accelerometer measurements [57, 58] -Measured physical fitness using handgrip strength, chair stand, 2-min step, back scratch, sit and reach, and flamingo balance test [57, 58] -Short Physical Performance Battery (SPPB) measures physical functioning using gait speed, standing balance, and lower leg strength [67]	

### Barriers and facilitators associated with co-design

We grouped author-reported barriers and facilitators into 5 categories, including framework and methodologies (any theoretical frameworks or principles used to formulate the co-design process and/or study methodology), logistics (details surrounding how the co-design process was organized and executed), relationships (dynamics among participants, and between participants and researchers), participation (participant engagement in the co-design process and the efforts made by researchers to increase engagement), and generalizability (aspects of the co-design process that help to make the intervention more applicable to the target population) (Table 3).

**Table 3:** Barriers and facilitators associated with co-design

Categories	Barriers	Facilitators
Frameworks & Methodologies	• Lack of literature on co-creation governance and frameworks [53] • Requiring participants to complete interventions before participating in co-design [68] • Complexity of data collection measures [36]	• Combining appreciative action and reflection, or an integrated knowledge translation approach with normalization process theory [50, 62] • User experience honeycomb model [43] • Participatory action research [51] • Photo-elicitation [69] • Training workshop and focus group facilitators in co-creation [52] • Fieldwork tasks [53] • Involving participants early, frequently, and throughout various stages [55, 59]
Logistics	• Open-ended questions [60] • Novice facilitators [60] • Hypothetical scenarios [39, 60] • Strategies to ensure members who belong to minority or socially disadvantaged groups are time intensive [57]	• Role and workshop aim clarification [53, 55, 62] • Debrief sessions for facilitators [62] • Sharing workshop summaries with participants [43, 52] • Utilizing community organizations or creating community advisory boards to assist with recruitment [51, 57] • Formal committee name and constitution [56]
Relationships	• Time and resources required to build trust within community of interest [57] • Bias arises from a desire to please and maintain group dynamics [36, 46] • Participatory Action Research approach may create a power differential [51] • Seniors require longer interviews [36]	• Building group dynamics (meeting in person, allowing time to socialize, and demonstrating appreciation for participation) [38, 55, 59]
Participation	• Fatigue/loss of concentration [39] • Participants who are more physically active may develop a louder voice than inactive participants taking away the perspective of the target population [37] • Cognitive, sensory, or physical disabilities may hinder participation [46] • Administrative costs associated with ongoing involvement [56] • Unfamiliarity with technology [47]	• Short sessions to prevent fatigue [36] • Homework tasks [62] • Small groups [61] • Comfortable location [57] • Active facilitator involvement [36, 37] • Assisting older adults to fill out questionnaires [36] • Increasing ownership of project [53, 64]
Generalizability	• Small sample size [50, 61] • Volunteers are more outspoken and active members of the community [57] • Variation in resources between communities [65]	• Purposive sampling [43] • Recruiting both experienced and novice technology users [63]

## Discussion

This is the first scoping review to map the body of existing literature on co-design and PA in older adults, and to summarize how and when co-design is implemented. We identified 29 unique studies that reported on the use of co-design to create PA interventions for older adults. Three key findings emerged from the review process. First, there is substantial variability in the terminology and operational definitions used to describe co-design and we propose a standardized definition based on common elements in the literature. Next, most studies used co-design during the conducting phase of research, less in the planning phase, and none involved end-users during the dissemination phase. Lastly, no studies directly evaluated the success or effectiveness of their co-design approach. The findings of our review have practical implications for the design of PA interventions in older adults.

Our study identified substantial variability in terminology and operational definitions amongst studies. The lack of a single consistent conceptualization of co-design is not unique to research in geriatric health promotion. It has been recognized as a challenge in other health areas of co-design research in terms of developing a comprehensive search strategy and trying to synthesize the literature in order to advance this area of knowledge [2, 70, 71]. Although we could not recommend a single unifying term, we propose a unifying definition that incorporates common thematic elements from the literature. Based on our findings, we define co-design as a user-centered approach involving collaboration between researchers, end-users, and other relevant stakeholders who are actively engaged throughout a process of iteration and continuous reflection to create an intervention tailored to the specific needs of the target population. Interestingly, while only one study included all components of our proposed definition in their operational definition, 21 studies included at least four of the 5 key components in their methods.

With respect to phases of involvement, most studies used co-design in the conducting phase, but none engaged end-users in the dissemination process. This is a noteworthy finding, as experts recommend the use of targeted dissemination to ensure maximal uptake [24, 25, 35]. Engaging end-users in dissemination may lead to more meaningful engagement of a wider range of people in the community, which could positively influence the uptake of evidence. Only one study involved end-users in consumer control. End-users should be involved throughout all phases of research to increase ownership of the findings among members of the public, who may then be more likely to share them within their social and community networks [35]. Potential reasons for the lack of higher levels of involvement include lack of funding, time and resources, and lack of implementation from healthcare staff, as has been reported by Brett et al., Donetto et al., and Baldwin et al. [5, 24, 25]. End-users can be involved in the dissemination process in many ways, such as seeking their opinions on which avenues should be used promote research findings or involving them in the development of tailored messaging to a wider audience.

Studies included in our review evaluated the co-design process indirectly by assessing outcomes downstream of the co-design process, such as the success of the PA intervention created during the co-design process, including PA, adherence, and participant satisfaction measures. These methods of evaluation are helpful to assess intervention adherence and to gauge participant satisfaction with the intervention or product, however, they do not allow evaluation of the co-design process itself. Despite all studies reporting positive outcomes of the PA intervention themselves, no study evaluated the success and/or effectiveness of the co-design process using direct methods, such as qualitatively assessing whether participants’ views were accurately represented in the final intervention. A possible reason for this is the observable gap in the literature may be related to a lack of standardized ways to evaluate the co-design process [2, 72]. Leask et al. recommends that evaluation be embedded throughout the phases of development to ensure that the intervention is representative of end-users’ ideas and tailored to their specific needs and circumstance [72]. Esmail and colleagues make similar recommendations and further suggest using external evaluators to minimize bias [73]. Additional strategies could include member checking or respondent validation [74, 75]. Another approach is to conduct a process evaluation of the intervention through assessment of facilitators and barriers of implementation, fidelity, and reach [76].

### Future research and implications

The field of co-design lacks a systematic framework to develop rigorous public health interventions and evaluate their efficacy and impact on a larger scale [72]. Leask et al. aimed to outline recommendations and key elements for the application and evaluation of co-created public health interventions using existing frameworks and methodologies and suggest models for increasing the scale of interventions to a population level [72]. We propose using common terminology and the operational definition proposed by our group to adopt a common language in this area of research. We recommend that future studies consider reporting their co-design interventions according to the guidelines by Leask et al. to improve the interpretation, replicability, and to guide the design of new studies [72].

Future research involving co-design and PA may benefit from knowledge of existing barriers identified in our review, which can be addressed in advance of the conducting phase. For example, researchers can develop strategies to increase accessibility of workshops to visible minorities and persons with disabilities, as well as investing in the training of group facilitators.

Our review further highlights the barriers and facilitators associated with the co-design process and supports existing literature in this area [25]. The barriers and facilitators summarized in this review can be used by researchers alongside recommendations by Leask et al. to design, implement, and evaluate co-designed interventions.

### Strengths and limitations

Our study has some limitations. We were only able to include studies published in English for feasibility. We recognize that important studies may be published in other languages that could contribute further to this review and our understanding of the co-design literature, however given the breadth of included studies, we feel we were able to retrieve a representative sample of the literature. Our study focused on the older adult population; therefore, our findings are not necessarily applicable to other age groups. Lastly, our search strategy did not include grey literature, however, there are no central sources for grey literature leading to challenges in locating relevant citations and a high probability of selection bias.

Our study also has important strengths. We developed a comprehensive search strategy that was peer-reviewed by two health research librarians. We included a large number of studies from various countries, which contributes to our understanding of co-designed PA interventions on a global scale. Similarly, we included a range of study designs, which allowed us to provide a more comprehensive summary of the existing evidence base. We also used an established scoping review protocol and registered our study on Open Science Framework to limit publication bias.

## Conclusion

Co-design is a growing and important area of research with substantial heterogeneity. This review mapped the co-design process for PA interventions in older adults, identifying gaps in when co-design is used and the level of involvement of end-users. The existing gaps in this body of research include the use co-design in the dissemination phase of research and systematic ways to assess the effectiveness of a co-designed intervention. Based on the included studies, we have suggested a standard definition of co-design for researchers to use moving forwards. Increasing the use of more standardized co-design methods presents an untapped potential for improving PA behaviour interventions. This review can help inform future co-designed interventions in their design and involvement of end-users to enhance the rigor and success of the process.

## Supplementary Information

**Additional file 1.** Preferred Reporting Items for Systematic Reviews and Meta-Analyses Extension for Scoping Review Checklist.

**Additional file 2.** Final search strategies for MEDLINE, AMED, EMBASE, and CINAHL Preferred Reporting Items for Systematic Reviews and Meta-analyses extension for scoping review.

**Additional file 3.** Preferred Reporting Items for Systematic Reviews and Meta-Analyses Flow Chart.

**Additional file 4.** Reference list of excluded studies and reasons for exclusion.

**Additional file 5.** Characteristics of included studies.

**Additional file 6.** Operational definitions of co-design terminology extracted.

## Data Availability

Not applicable as this is a scoping review and therefore included no primary data collection.

## Abbreviations

WHO: World Health Organization
PA: Physical activity
JBI: Joanna Briggs Institute
PRISMA: Preferred Reporting Items for Systematic Reviews
PCORI: Patient-Centered Outcomes Research Institute
AMED: Allied and Complementary Medicine database
CINAHL: Cumulative Index to Nursing and Allied Health Professionals
PRISMA-ScR: Preferred Reporting Items for Systematic Reviews and Meta-Analyses Extension for Scoping Review
