# Supplementary material for: The use of co-design in developing physical activity interventions for older adults: a scoping review
Source: BMC Geriatr. 2022 Aug 8;22:647. doi: 10.1186/s12877-022-03345-4 (PMC9358386; doi:10.1186/s12877-022-03345-4)
Supplement: Supplementary file 2 — Additional file 2. Final search strategies for MEDLINE, AMED, EMBASE, and CINAHL Preferred Reporting Items for Systematic Reviews and Meta-analyses extension for scoping review. [file 12877_2022_3345_MOESM2_ESM.docx]

Additional File 2: Search Strategy

**Final Search Strategies for MEDLINE, AMED, EMBASE, and CINAHL**

All search strategies were reviewed and approved by our supervisors as well as two Health Science Librarians, Ms. Neera Bhatnagar and Mr. Jack Young.

**Database:** MEDLINE

**Date of search:** February 18^th^, 2021

**Limits:** No limits were applied to this search strategy.

**Operators**: all mesh headings and key words under the same construct (i.e., physical activity) were combined with the operator “OR” and all constructs were combined with the operator “AND”.

**Number of studies retrieved:** 7,377

| Construct | Mesh Headings | Key words |
| --- | --- | --- |
| Physical Activity | Exercise (exp)  Exercise therapy (exp)  Recreation (exp)  Recreation therapy (exp)  Motor activity (exp)  Movement (exp)  Sports (exp)  Sports equipment (exp)  Activities of daily living (exp)  Physical Fitness (exp)  Walking (exp)  Exercise movement techniques (exp)  Physical endurance (exp) | Abdominal exercis*  Activit* of daily living  Aerobic exercis*  Anaerobic exercis*  Arm exercis*  Aquatic exercis*  Breathing exercis*  Cardiorespiratory fitness  Closed kinetic chain exercis*  Danc*  Dynamic exercis*  Exercis*  Exercise and fitness equipment  Exercis* position  Exercis* intensity  Exercis* therapy  Fitness  Group exercis*  Isokinetic exercis*  Isotonic exercis*  Isometric exercis*  Kegel exercis*  Leg exercis*  Moderate-vigorous physical activ*  Motor activit*  Movement techniqu*  Movement  Muscle exercis*  Open kinetic chain exercis*  Physical fit*  Physical activ*  Plyometric*  Recreation  Resistance training  Sport*  Sport*equipment  Static exercis*  Stretching exercis*  Swim*  Treadmill exercis*  Therapeutic exercis*  Walk*  Warm-up exercis* |
| Co-design | Community-based participatory research (exp)  Patient Participation (exp)  Community participation (exp) | Action research  Advisory  Co-creat*  Cocreat*  Co-design  Codesign  Co-produc*  Coproduc*  Co-evaluat*  Coevaluat*  Co-decision*  Codecision*  Co-method*  Community-based participatory research  Community participation  Engagement  Participatory design  Participatory research  Participatory health research  Patient participation  Public and patient involvement  User involvement |
| Older adults | Aged (exp)  Aging (exp) | Aged  Aged individual*  Aged hospital patient*  Aged patient*  Aged worker*  Age 60  Age 65  Age 70  Age 75  Age 80  Aging adult*  Elder*  Frail elderly  Geriatric*  Late life  Master* athlete*  Old* adult*  Old* patient*  Old* people  Old* veteran*  Senior*  Very elderl*  60 and over |

**Database:** AMED

**Date of search:** February 18^th^, 2021

**Limits:** No limits were applied to this search strategy.

**Operators**: all mesh headings and key words under the same construct (i.e., physical activity) were combined with the operator “OR” and all constructs were combined with the operator “AND”.

**Number of studies retrieved:** 280

| Construct | Subject Heading | Key words |
| --- | --- | --- |
| Physical Activity | Exercise (exp)  Exercise therapy (exp)  Exercise movement techniques (exp)  Recreation (exp)  Motor activity (exp)  Movement (exp)  Sports (exp)  Sports equipment (exp)  Activities of daily living (exp)  Physical Fitness (exp)  Walking (exp)  Physical endurance (exp)  Rehabilitation (exp) | Abdominal exercis*  Activit* of daily living  Aerobic exercis*  Anaerobic exercis*  Arm exercis*  Aquatic exercis*  Breathing exercis*  Cardiorespiratory fitness  Closed kinetic chain exercis*  Danc*  Dynamic exercis*  Exercis*  Exercise and fitness equipment  Exercis* position  Exercis* intensity  Exercis* therapy  Fitness  Group exercis*  Isokinetic exercis*  Isotonic exercis*  Isometric exercis*  Kegel exercis*  Leg exercis*  Moderate to vigorous physical activ*  Motor activit*  Movement techniqu*  Movement  Muscle exercis*  Open kinetic chain exercis*  Physical fit*  Physical activ*  Plyometric*  Recreation  Resistance training  Sport*  Sport* equipment  Static exercis*  Stretching exercis*  Swim*  Treadmill exercis*  Therapeutic exercis*  Walk*  Warm-up exercis* |
| Co-design | Patient Participation (exp) | Action research  Advisory  Co-creat*  Cocreat*  Co-design  Codesign  Co-produc*  Coproduc*  Co-method*  Community-based participatory research  Community participation  Engagement  Participatory design  Participatory research  Patient participation  Public and patient involvement  User involvement  End-user involvement |
| Older adults | Aged (exp)  Aging (exp)  Frail elderly (exp)  Geriatrics (exp) | Aged  Aged individual*  Aged hospital patient*  Aged patient*  Aged worker*  Age 60  Age 65  Age 70  Age 75  Age 80  Aging adult*  Elder*  Frail elderly  Geriatric*  Late life  Master* athlete*  Old* adult*  Old* patient*  Old* people  Old* veteran*  Senior*  Very elder*  60 and over |

**Database:** EMBASE

**Date of search:** February 18^th^, 2021

**Limits:** No limits were applied to this search strategy.

**Operators**: all mesh headings and key words under the same construct (i.e., physical activity) were combined with the operator “OR” and all constructs were combined with the operator “AND”.

**Number of studies retrieved:** 5,944

| Construct | Mesh Headings | Key words |
| --- | --- | --- |
| Physical Activity | aerobic exercise (exp)  breathing exercise (exp)  exercise (exp)  isometric exercise (exp)  muscle exercise (exp)  anaerobic exercise (exp)  aquatic exercise (exp)  arm exercise (exp)  closed kinetic chain exercise (exp)  dynamic exercise (exp)  “exercise and fitness equipment” (exp)  isokinetic exercise (exp)  isotonic exercise (exp)  leg exercise (exp)  open kinetic chain exercise (exp)  static exercise (exp)  stretching exercise (exp)  treadmill exercise (exp)  physical activity (exp)  walking (exp)  swimming (exp)  dancing (exp)  sport (exp)  fitness (exp)  cardiorespiratory fitness (exp) | Aerobic exercis*  Breathing exercis*  Isometric exercis*  Muscle exercis*  Anaerobic exercis*  Aquatic exercis*  Arm exercis*  Closed kinetic chain exercis*  Dynamic exercis*  Exercise and fitness equipment  Isokinetic exercis*  Isotonic exercis*  Leg exercis*  Open kinetic chain exercis*  Static exercis*  Stretching exercis*  Treadmill exercis*  walk*  swim*  activit* of daily living  danc*  sport*  Fitness  Cardiorespiratory fitness  Movement techniques  Physical activ*  Exercis*  Physical fit*  Moderate-vigorous physical activ*  resistance training  exercis* position* |
| older adults | aged (exp)  aged hospital patient (exp)  aged worker (exp)  geriatrics (exp)  very elderly (exp) | Aged  Aged hospital patient*  Aged worker  Old* adult*  Elder*  Senior*  Master* athlete*  60 and over  aged patient*  aged individual*  old* patient*  old* people  old* veteran*  geriatric*  Very elderly  age 60  age 65  age 70  age 75  age 80  late life  Frail elderly  Older adult*  elderly  aging adult*  60 and over |
| co-design | participatory research (exp)  patient participation (exp)  community participation (exp) | Participatory research  Community based participatory research  Patient participation  Community participation  Co-design  Codesign  Co-creat*  Co-produc*  Coproduc*  Co-decision*  Codecision*  Participatory design  Participatory research  Participatory health research  Public and patient involvement  Action research  Engagement  Advisory  User involvement  Generative design research  Scandinavian Design Research  Community-based participatory research |

**Database:** CINAHL

**Date of search:** February 18^th^, 2021

**Limits:** No limits were applied to this search strategy.

**Operators**: all mesh headings and key words under the same construct (i.e., physical activity) were combined with the operator “OR” and all constructs were combined with the operator “AND”.

**Number of studies retrieved:** 2,452

| Construct | MESH terms | Key words |
| --- | --- | --- |
| Physical Activity | Physical activity (exp)  Exercise+ (exp)  resistance training (exp)  exercise positions+ (exp)  abdominal exercises (exp)  therapeutic exercise+ (exp)  warm-up exercise+ (exp)  Recovery, exercise (exp)  group exercise (exp)  sport specific training (exp)  kegel exercises (exp)  exercise intensity (exp)  open kinetic chain exercises (exp)  plyometrics (exp) | physical activit*  exercis*  resistance training  exercis* position*  Abdominal exercis*  Therapeutic exercis*  Warm-up exercis*  exercis* recovery  Group exercis*  Kegel exercis*  Sport specific training  exercis* intensity  Open kinetic chain exercis*  plyometric  Physical fit*  Moderate-vigorous physical activ*  Walk*  Swim*  Activit* of daily living  Danc*  Sport*  Fitness  Movement techniques  Exercise therapy  Recreation  Motor activity  Movement  Aerobic exercis*  Breathing exercis*  Isometric exercis*  Muscle exercis*  Anaerobic exercis*  Aquatic exercis*  Arm exercis*  Closed kinetic chain exercis*  Dynamic exercis*  Isokinetic exercis*  Isotonic exercis*  Leg exercis*  Static exercis*  Stretching exercis*  Treadmill exercis*  Cardiorespiratory fitness |
| Co-design | Action research (exp) | co-design  codesign  co-creat*  cocreat*  co-produc*  coproduc*  co-evaluat*  coevaluat*  co-decision  codecision  participatory design  participatory research  Participatory health research  Public and patient involvement  Action research  Patient participation  User involvement  Community participation  Engagement  Advisory  Generative design research  Scandinavian Design Research  Community based participatory research |
| Older adults | Frail elderly (exp)  aged+ (exp)  aged, 80 and over+ (exp)  aged, hospitalized (exp) | frail elderly  older adult*  geriatric*  aged  senior*  elderly  aging adult*  Old* adult*  Elder*  Master* athlete*  60 and over  aged patient*  aged individual*  old* patient*  old* people  old* veteran*  age 60  age 65  age 70  age 75  age 80  late life  Aged hospital patient*  Aged worker  Very elderly |
