# Supplementary material for: The use of co-design in developing physical activity interventions for older adults: a scoping review
Source: BMC Geriatr. 2022 Aug 8;22:647. doi: 10.1186/s12877-022-03345-4 (PMC9358386; doi:10.1186/s12877-022-03345-4)
Supplement: Supplementary file 3 — Additional file 3. Preferred Reporting Items for Systematic Reviews and Meta-Analyses Flow Chart. [file 12877_2022_3345_MOESM3_ESM.docx]

Additional File 3: Preferred Reporting Items for Systematic reviews and Meta-Analyses (PRISMA) Flow Chart
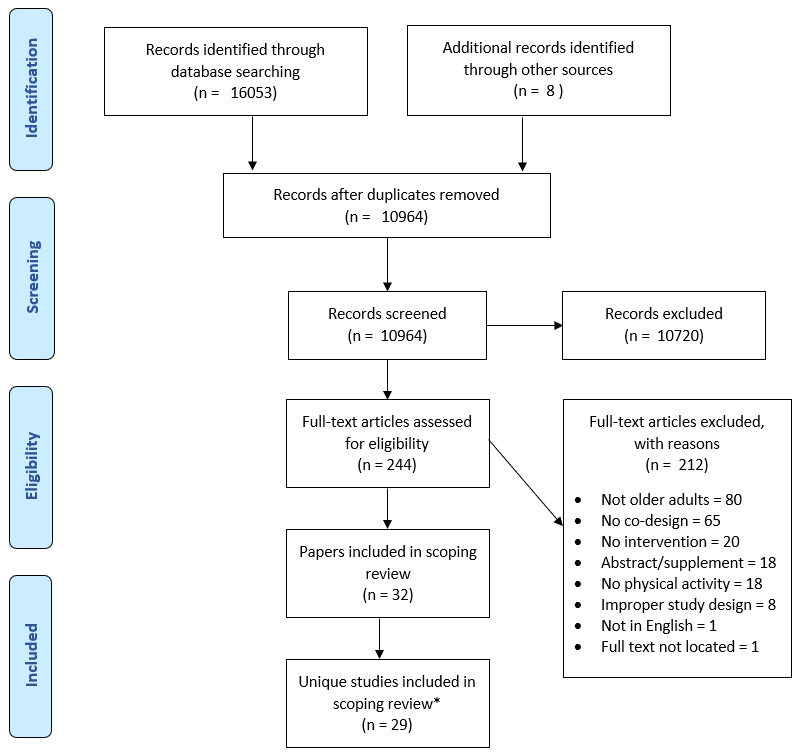


Reference: Moher D, Liberati A, Tetzlaff J, Altman DG, The PRISMA Group (2009). Preferred Reporting Items for Systematic Reviews and Meta Analyses: The PRISMA Statement. PLoS Med 6(7): e1000097. doi:10.1371/journal.pmed1000097 (37). *Three studies had secondary publications including a protocol paper and two qualitative papers associated with randomized controlled trials, leaving 29 unique studies.
