# Supplementary material for: The use of co-design in developing physical activity interventions for older adults: a scoping review
Source: BMC Geriatr. 2022 Aug 8;22:647. doi: 10.1186/s12877-022-03345-4 (PMC9358386; doi:10.1186/s12877-022-03345-4)
Supplement: Supplementary file 4 — Additional file 4. Reference list of excluded studies and reasons for exclusion. [file 12877_2022_3345_MOESM4_ESM.docx]

Additional File 4: Reference list of excluded studies and reasons for exclusion

| **Reference** | **Reason for Exclusion** |
| --- | --- |
| Zoellner J, Hill JL, Zynda K, Sample AD, Yadrick K. Environmental perceptions and objective walking trail audits inform a community-based participatory research walking intervention. The international journal of behavioral nutrition and physical activity. 2012;9(101217089):6. | Not older adults |
| Zaragoza Casterad J, Sevil-Serrano J, Bois JE, Generelo E, Lhuisset L, Aibar-Solana A. Centre for the Promotion of Physical Activity and Health (CAPAS-City): A Pyrenean Cross-Cultural Structure to Lead the Way in the Design, Implementation, and Evaluation of Multilevel Physical Activity Interventions. International journal of environmental research and public health. 2019;16(19). | Not older adults |
| Yazdanpanah B., Safari M., Angha P., Karami M., Emadi M., Yazdanpanah S., et al. Efficacy of community-based participatory research on diabetes care in Yasouj-Iran. Iranian Journal of Epidemiology. 2012;7(4):1–8. (Persian) | Not in English |
| Yardley L., Beyer N., Hauer K., McKee K., Ballinger C., Todd C. Recommendations for promoting the engagement of older people in activities to prevent falls. Quality and Safety in Health Care. 2007;16(3):230–4. | No co-design |
| Wynn TA, Wyatt SB, Hardy CM, Walker SS, Thomas TF, Williams AG, et al. Using Community Feedback to Improve Community Interventions: Results From the Deep South Network for Cancer Control Project. Family & community health. 2016;39(4):234–41. | No physical activity |
| Wu Z, Xu J, Yue C, Li Y, Liang Y. Collaborative Care Model Based Telerehabilitation Exercise Training Program for Acute Stroke Patients in China: A Randomized Controlled Trial. Journal of stroke and cerebrovascular diseases : the official journal of National Stroke Association. 2020;29(12):105328. | Not older adults |
| Woods L, Roehrer E, Duff J, Walker K, Cummings E. Co-Design of a Mobile Health App for Heart Failure: Perspectives from the Team...Health Informatics Conference, August 12-14, 2019, Melbourne, Australia. Studies in Health Technology & Informatics. 2019;266:183–8. | No physical activity |
| Wong EY-S, Lee AH, James AP, Jancey J. Physical activity and nutrition intervention for Singaporean women aged 50 years and above: study protocol for a randomised controlled trial. Trials. 2018;19(1):257. | No co-design |
| Wolever RQ, Dreusicke M, Fikkan J, Hawkins TV, Yeung S, Wakefield J, et al. Integrative health coaching for patients with type 2 diabetes: a randomized clinical trial. The Diabetes educator. 2010;36(4):629–39. | Not older adults |
| Woda A, Haglund K, Belknap RA, Sebern M. Self-Care Behaviors of African Americans Living with Heart Failure. Journal of community health nursing. 2015;32(4):173–86. | No physical activity |
| Withall J, Thompson JL, Fox KR, Davis M, Gray S, Koning J de, et al. Participant and Public Involvement in Refining a Peer-Volunteering Active Aging Intervention: Project ACE (Active, Connected, Engaged). Gerontologist. 2018;58(2):362–75. | No co-design |
| Williams SL, McSharry J, Taylor C, Dale J, Michie S, French DP. Translating a walking intervention for health professional delivery within primary care: A mixed-methods treatment fidelity assessment. British journal of health psychology. 2020;25(1):17–38. | Not older adults |
| Williams L II, Olano VR. Mobilizing and maintaining a coalition to promote physical activity among African Americans in Southeast Stockton, California. Journal of Health Education. 1999;30(2):S31-6. | No co-design |
| Williams GR, Nyrop KA, Deal AM, Muss HB, Sanoff HK. Self-directed physical activity intervention in older adults undergoing adjuvant chemotherapy for colorectal cancer: Design of a randomized controlled trial. Contemporary clinical trials. 2015;42(101242342):90–7. | Not older adults |
| Wilcox S, Laken M, Bopp M, Gethers O, Huang P, McClorin L, et al. Increasing physical activity among church members: community-based participatory research. American journal of preventive medicine. 2007;32(2):131–8. | Not older adults |
| Whitney J., Skinner L., Brown H., Hurley M. Service users and providers perspectives of an exercise-based falls prevention programme for people with cognitive impairment. Physiotherapy (United Kingdom). 2011;97(SUPPL. 1):eS1342–3. | Abstract or poster |
| Whipple MO, Talley KMC, Schorr E, Bronas UG, Treat-Jacobson D. Patterns of Physical Activity Attitudes and Engagement in Physical Activity among Older Adults with Peripheral Artery Disease and Diabetes: A Mixed Methods Study. Journal of Vascular Nursing. 2018;36(2):104–104. | No co-design |
| Whipple MO, Schorr EN, Talley KMC, Lindquist R, Bronas UG, Treat-Jacobson D. A mixed methods study of perceived barriers to physical activity, geriatric syndromes, and physical activity levels among older adults with peripheral artery disease and diabetes. Journal of vascular nursing : official publication of the Society for Peripheral Vascular Nursing. 2019;37(2):91–105. | No co-design |
| Wang H.-W., Shi C.R., Keir G. An integrated tai chi intervention for fall prevention and wellness promotion among elderly patients at bowdoin street health center. Journal of General Internal Medicine. 2015;30(SUPPL. 2):S523–4. | Abstract/supplement |
| Wang E.Y., Graff R.E., Chan J.M., Langlais C.S., Broering J.M., Ramsdill J.W., et al. Web-based lifestyle interventions for prostate cancer survivors: Qualitative study. JMIR Cancer. 2020;6(2):e19362. | No co-design |
| Walsh DM, Moran K, Cornelissen V, Buys R, Cornelis N, Woods C. Electronic Health Physical Activity Behavior Change Intervention to Self-Manage Cardiovascular Disease: Qualitative Exploration of Patient and Health Professional Requirements. Journal of medical Internet research. 2018;20(5):e163. | No co-design |
| Verbiest M, Borrell S, Dalhousie S, Tupa’i-Firestone R, Funaki T, Goodwin D, et al. A Co-Designed, Culturally-Tailored mHealth Tool to Support Healthy Lifestyles in Maori and Pasifika Communities in New Zealand: Protocol for a Cluster Randomized Controlled Trial. JMIR research protocols. 2018;7(8):e10789. | Not older adults |
| Veenhof C., van Hasselt T.J., Koke A.J.A., Dekker J., Bijlsma J.W.J., van den Ende C.H.M. Active involvement and long-term goals influence long-term adherence to behavioural graded activity in patients with osteoarthritis: A qualitative study. Australian Journal of Physiotherapy. 2006;52(4):273–8. | No co-design |
| Van Leuven KA. Health practices of older adults in good health engagement is the key. Journal of Gerontological Nursing. 2010;36(6):38–46. | No intervention |
| Vahabi M, Damba C. A feasibility study of a culturally and gender-specific dance to promote physical activity for South Asian immigrant women in the greater Toronto area. Women’s health issues : official publication of the Jacobs Institute of Women’s Health. 2015;25(1):79–87. | Not older adults |
| Tung H-T, Chen K-M, Yao C-T, Kuo C-C, Hsu H-F. Self-evaluation by community older adults on the applicability of the healthy beat acupunch exercise program. Complementary Therapies in Medicine. 2019;42:59–64. | No co-design |
| Tudor-Locke C, Jones R, Myers AM, Paterson DH, Ecclestone NA. Contribution of structured exercise class participation and informal walking for exercise to daily physical activity in community-dwelling older adults. 2002;73(3):350–6. | No co-design |
| Tong A, Tunnicliffe DJ, Lopez-Vargas P, Mallett A, Patel C, Savige J, et al. Identifying and integrating consumer perspectives in clinical practice guidelines on autosomal-dominant polycystic kidney disease. Nephrology (Carlton, Vic). 2016;21(2):122–32. | Not older adults |
| Tong A, Lopez-Vargas P, Howell M, Phoon R, Johnson D, Campbell D, et al. Consumer involvement in topic and outcome selection in the development of clinical practice guidelines. Health expectations : an international journal of public participation in health care and health policy. 2012;15(4):410–23. | Not older adults |
| Tischler V, Schneider J, Morgner C, Crawford P, Dening T, Brooker D, et al. Stronger together: learning from an interdisciplinary dementia, arts and well-being network (DA&WN). Arts & health. 2019;11(3):272–7. | No co-design |
| Thiamwong L, Stout JR, Sole ML, Ng BP, Yan X, Talbert S. Physio-Feedback and Exercise Program (PEER) Improves Balance, Muscle Strength, and Fall Risk in Older Adults. Research in gerontological nursing. 2020;13(6):289–96. | No co-design |
| Takano T, Nakamura K. Participatory research to enhance vision sharing for Healthy Town initiatives in Japan. Health promotion international. 2004;19(3):299–307. | No co-design |
| Stickney B, Vilshanskaya O. Engaging older people with English as a second language and frail older people in physical activity. Health promotion journal of Australia : official journal of Australian Association of Health Promotion Professionals. 2005;16(2):116–23. | No co-design |
| Stevens AB, Thiel SB, Thorud JL, Smith ML, Howell D, Cargill J, et al. Increasing the Availability of Physical Activity Programs for Older Adults: Lessons Learned From Texercise Stakeholders. Journal of aging and physical activity. 2016;24(1):39–44. | No co-design |
| Spoelstra SL, Sikorskii A, Gitlin LN, Schueller M, Kline M, Szanton SL. Dissemination of the CAPABLE Model of Care in a Medicaid Waiver Program to Improve Physical Function. Journal of the American Geriatrics Society. 2019;67(2):363–70. | No co-design |
| Solem I.K.L., Varsi C., Eide H., Kristjansdottir O.B., Borosund E., Schreurs K.M.G., et al. A user-centered approach to an evidence-based electronic health pain management intervention for people with chronic pain: Design and development of EPIO. Journal of Medical Internet Research. 2020;22(1):e15889. | Not older adults |
| Smith CM, Hale LA, Mulligan HF, Treharne GJ. Participant perceptions of a novel physiotherapy approach (‘Blue Prescription’) for increasing levels of physical activity in people with multiple sclerosis: a qualitative study following intervention. Disability and rehabilitation. 2013;35(14):1174–81. | Not older adults |
| Short CE, Finlay A, Sanders I, Maher C. Development and pilot evaluation of a clinic-based mHealth app referral service to support adult cancer survivors increase their participation in physical activity using publicly available mobile apps. BMC health services research. 2018;18(1):27. | Not older adults |
| Shiner B, Whitley R, Van Citters AD, Pratt SI, Bartels SJ. Learning what matters for patients: qualitative evaluation of a health promotion program for those with serious mental illness. Health promotion international. 2008;23(3):275–82. | Not older adults |
| Sheerin F., Lynch A., Kilroy S., Epstein M., Girault A., Larose I., et al. A qualitative study exploring facilitators and barriers to engagement in physical activity among older Irish and French adults. Age and Ageing. 2019;48(Supplement 3). | Improper study design |
| Shanahan J, Bhriain ON, Morris ME, Volpe D, Clifford AM. Irish set dancing classes for people with Parkinson’s disease: The needs of participants and dance teachers. Complementary therapies in medicine. 2016;27(9308777, c6k):12–7. | No co-design |
| Seguin-Fowler R, Graham M, Sriram U, Eldridge G, Kim J, Tom M. Web-Based Dissemination of a Civic Engagement Curriculum to Promote Healthy Eating and Active Living in Rural Towns: The eHEART Study. International journal of environmental research and public health. 2020;17(7). | Not older |
| Seguin RA, Eldridge G, Graham ML, Folta SC, Nelson ME, Strogatz D. Strong Hearts, healthy communities: a rural community-based cardiovascular disease prevention program. BMC public health. 2016;16(100968562):86. | No co-design |
| Sebastiao E, Ibe-Lamberts K, Bobitt J, Schwingel A, Chodzko-Zajko W. Employing a Participatory Research Approach to Explore Physical Activity among Older African American Women. Journal of aging research. 2014;2014(101543460):941019. | No intervention |
| Sebastiao E, Chodzko-Zajko W, Schwingel A. An In-Depth Examination of Perceptions of Physical Activity in Regularly Active and Insufficiently Active Older African American Women: A Participatory Approach. PloS one. 2015;10(11):e0142703. | No intervention |
| Schulz AJ, Israel BA, Mentz GB, Bernal C, Caver D, DeMajo R, et al. Effectiveness of a walking group intervention to promote physical activity and cardiovascular health in predominantly non-Hispanic black and Hispanic urban neighborhoods: findings from the walk your heart to health intervention. Health education & behavior : the official publication of the Society for Public Health Education. 2015;42(3):380–92. | Not older adults |
| Schulz A.J., Zenk S., Odoms-Young A., Hollis-Neely T., Nwankwo R., Lockett M., et al. Healthy eating and exercising to reduce diabetes: Exploring the potential of social determinants of health frameworks within the context of community-based participatory diabetes prevention. American Journal of Public Health. 2005;95(4):645–51. | Not older adults |
| Schroeder K, Ratcliffe SJ, Perez A, Earley D, Bowman C, Lipman TH. Dance for Health: An Intergenerational Program to Increase Access to Physical Activity. Journal of pediatric nursing. 2017;37(jns, 8607529):29–34. | Not older adults |
| Schroeder K, Deatrick JA, Klusaritz H, Bowman C, Williams TT, Lee J, et al. Using a Community Workgroup Approach to Increase Access to Physical Activity in an Underresourced Urban Community. Health promotion practice. 2020;21(1):5–11. | Not older adults |
| Scandurra I, Sjolinder M. Participatory Design With Seniors: Design of Future Services and Iterative Refinements of Interactive eHealth Services for Old Citizens. Medicine 20. 2013;2(2):e12. | No physical activity |
| Sayer J, Paniagua D, Ballentine S, Sheehan L, Carson M, Nieweglowski K, et al. Perspectives on diet and physical activity among urban African Americans with serious mental illness. Social work in health care. 2019;58(5):509–25. | Not older adults |
| Sarobol S. Participatory action research on public health policy development for physical activities in local administration organizations, Chiang Mai Province, Thailand. Journal of Science and Medicine in Sport. 2012;15(SUPPL.1):S322–3. | Abstract/supplement |
| Salvatore AL, Noonan CJ, Williams MB, Wetherill MS, Jacob T, Cannady TK, et al. Social Support and Physical Activity Among American Indians in Oklahoma: Results From a Community-based Participatory Research Study. The Journal of rural health : official journal of the American Rural Health Association and the National Rural Health Care Association. 2019;35(3):374–84. | Not older adults |
| Sabin K.L. Older adults and motivation for therapy and exercise: Issues, influences, and interventions. Topics in Geriatric Rehabilitation. 2005;21(3):215–20. | No intervention |
| Revenas A, Opava CH, Asenlof P. Lead users’ ideas on core features to support physical activity in rheumatoid arthritis: a first step in the development of an internet service using participatory design. BMC medical informatics and decision making. 2014;14(101088682):21. | Not older adults |
| Buckley BJR, Thijssen DHJ, Murphy RC, Graves LEF, Whyte G, Gillison FB, et al. Making a move in exercise referral: co-development of a physical activity referral scheme. Journal of Public Health. 2018;40(4):e586–93. | Not older adults |
| Reininger BM, Barroso CS, Mitchell-Bennett L, Cantu E, Fernandez ME, Gonzalez DA, et al. Process evaluation and participatory methods in an obesity-prevention media campaign for Mexican Americans. Health promotion practice. 2010;11(3):347–57. | Not older adults |
| Razai MS, Oakeshott P. The Importance of Community and Patient Involvement in the Design of Physical Activity Programs. Journal of Primary Care & Community Health. 2020;11:1–1. | Not older adults |
| Ray A.D., Masucci Twarozek A., Williams B.T., Erwin D.O., Underwood W., Mahoney M.C. Exercise in african American and white colorectal cancer survivors: A mixed-methods approach. Rehabilitation Oncology. 2018;36(4):188–97. | Not older adults |
| Rasche P, Mertens A, Brandl C, Liu S, Buecking B, Bliemel C, et al. Satisfying Product Features of a Fall Prevention Smartphone App and Potential Users’ Willingness to Pay: Web-Based Survey Among Older Adults. JMIR mHealth and uHealth. 2018;6(3):e75. | No co-design |
| Radhakrishnan K, Julien C, O’Hair M, Baranowski T, Lee G, Allen C, et al. Usability Testing of a Sensor-Controlled Digital Game to Engage Older Adults with Heart Failure in Physical Activity and Weight Monitoring. Applied clinical informatics. 2020;11(5):873–81. | No co-design |
| Radford K., Delbaere K., Garvey G., Lavrencic L., Donovan T., Allan W., et al. Dementia Prevention Programs In Urban And Regional Aboriginal Communities In Australia. Alzheimer’s and Dementia. 2019;15(7 Supplement):P174. | Abstract/supplement |
| Perry CK, Weatherby K. Feasibility of an Intergenerational Tai Chi Program: A Community-Based Participatory Research Project. Journal of Intergenerational Relationships. 2011;9(1):69–84. | Not older adults |
| Perry CK, McCalmont JC, Ward JP, Menelas H-DK, Jackson C, De Witz JR, et al. Mujeres Fuertes y Corazones Saludables: adaptation of the StrongWomen -healthy hearts program for rural Latinas using an intervention mapping approach. BMC public health. 2017;17(1):982. | Not older adults |
| Pedroli E, Greci L, Colombo D, Serino S, Cipresso P, Arlati S, et al. Characteristics, Usability, and Users Experience of a System Combining Cognitive and Physical Therapy in a Virtual Environment: Positive Bike. Sensors (Basel, Switzerland). 2018;18(7). | No co-design |
| Pazoki R, Nabipour I, Seyednezami N, Imami SR. Effects of a community-based healthy heart program on increasing healthy women’s physical activity: a randomized controlled trial guided by Community-based Participatory Research (CBPR). BMC public health. 2007;7(100968562):216. | Not older adults |
| Pawlowski CS, Winge L, Carroll S, Schmidt T, Wagner AM, Nortoft KPJ, et al. Move the Neighbourhood: Study design of a community-based participatory public open space intervention in a Danish deprived neighbourhood to promote active living. BMC public health. 2017;17(1):481. | No physical activity |
| Papageorgiou N, Marquis R, Dare J. Identifying the enablers and barriers to community participation amongst older adults. British Journal of Occupational Therapy. 2016;79(12):742–51. | No intervention |
| Ostlund-Lagerstrom L, Blomberg K, Algilani S, Schoultz M, Kihlgren A, Brummer RJ, et al. Senior orienteering athletes as a model of healthy aging: a mixed-method approach. BMC geriatrics. 2015;15(100968548):76. | No co-design |
| Oba N, McCaffrey R, Choonhapran P, Chutug P, Rueangram S. Development of a community participation program for diabetes mellitus prevention in a primary care unit, Thailand. Nursing & health sciences. 2011;13(3):352–9. | Not older adults |
| Nykiforuk CIJ, Coupland K, Nieuwendyk LMJ, Ann McGetrick J. Universal Design for the rural walks of life: operationalizing walkability in Bonnyville, Alberta, Canada. Critical Public Health. 2018;28(2):213–24. | Not older adults |
| Niles B.L., Mori D.L., Polizzi C.P., Kaiser A.P., Ledoux A.M., Wang C. Feasibility, qualitative findings and satisfaction of a brief Tai Chi mind-body programme for veterans with post-traumatic stress symptoms. BMJ Open. 2016;6(11):e012464. | Not older adults |
| Ni Mhurchu C, Te Morenga L, Tupai-Firestone R, Grey J, Jiang Y, Jull A, et al. A co-designed mHealth programme to support healthy lifestyles in Maori and Pasifika peoples in New Zealand (OL@-OR@): a cluster-randomised controlled trial. The Lancet Digital health. 2019;1(6):e298–307. | Not older adults |
| Nery-Hurwit M, Kincl L, Driver S, Heller B. Stakeholder evaluation of an online program to promote physical activity and workplace safety for individuals with disability. Evaluation and program planning. 2017;63(7801727, eob):39–44. | Not older adults |
| Neil-Sztramko SE, Smith-Turchyn J, Richardson J, Dobbins M. A Mobility-Focused Knowledge Translation Randomized Controlled Trial to Improve Physical Activity: Process Evaluation of the Move4Age Study. Journal of medical Internet research. 2019;21(6):e13965. | No co-design |
| Nau T, Nolan G, Smith BJ. Enhancing Engagement With Socially Disadvantaged Older People in Organized Physical Activity Programs. International quarterly of community health education. 2019;39(4):257–67. | No co-design |
| Naik H., Liu G., Qiu X., Pringle D., Brown C., Eng L., et al. Effect of physical activity (PA) perceptions in cancer survivors on PA behaviors: Helping health care providers improve patient communication. Journal of Clinical Oncology [Internet]. 2014;32(30 SUPPL. 1). Available from: http://meeting.ascopubs.org/cgi/content/abstract/32/30_suppl/201?sid=12d55207-fb62-4557-9a71-127c2f4d67bf | Abstract/supplement |
| Myhre Jensen C, Overgaard S, Wiil UK, Clemensen J. Telemedicine, as a way to qualify the orthopaedic pathway - a Participatory Design study. International Journal of Integrated Care (IJIC). 2016;16(5 Supplement):1–3. | No physical activity |
| Murdoch-Flowers J, Tremblay M-C, Hovey R, Delormier T, Gray-Donald K, Delaronde E, et al. Understanding how Indigenous culturally-based interventions can improve participants’ health in Canada. Health promotion international. 2019;34(1):154–65. | Not older adults |
| Mulligan H, Treharne GJ, Hale LA, Smith C. Combining Self-help and Professional Help to Minimize Barriers to Physical Activity in Persons With Multiple Sclerosis. Journal of Neurologic Physical Therapy. 2013;37(2):51–7. | Not older adults |
| Motl RW, Backus D, Neal WN, Cutter G, Palmer L, McBurney R, et al. Rationale and design of the STEP for MS Trial: Comparative effectiveness of Supervised versus Telerehabilitation Exercise Programs for Multiple Sclerosis. Contemporary clinical trials. 2019;81(101242342):110–22. | Not older adults |
| Morrison J, Akter K, Jennings HM, Nahar T, Kuddus A, Shaha SK, et al. Participatory learning and action to address type 2 diabetes in rural Bangladesh: a qualitative process evaluation. BMC endocrine disorders. 2019;19(1):118. | Not older adults |
| Morris J.H., Oliver T., Kroll T., Joice S., Williams B. Physical activity participation in community dwelling stroke survivors: synergy and dissonance between motivation and capability. A qualitative study. Physiotherapy (United Kingdom). 2017;103(3):311–21. | No intervention |
| Moore A.P., Stanton-Fay S.H., Rivas C.A., Harding S., Goff L.M. Co-design of a culturally-tailored diet & lifestyle intervention for diabetes management in the UK African-Caribbean community. Proceedings of the Nutrition Society. 2017;76(OCE4). | No intervention |
| Mogle J, Lorek A, Dattilo J, Freed S, Frysinger M, Schuckers S. Activity Engagement in Community Dwelling Older Adults: Motivations, Barriers, and Success...28th Annual Scientific Session, June 2-6, 2017, Baltimore, Maryland. Nursing Research. 2016;65(2):E97–E97. | No intervention |
| Menkin JA, McCreath HE, Song SY, Carrillo CA, Reyes CE, Trejo L, et al. ‘Worth the Walk’: Culturally Tailored Stroke Risk Factor Reduction Intervention in Community Senior Centers. Journal of the American Heart Association. 2019;8(6):e011088. | No co-design |
| Mendoza-Nunez VM, Vivaldo-Martinez M. Community Gerontology Model for Healthy Aging Developed in Mexico Framed in Resilience and Generativity. Journal of cross-cultural gerontology. 2019;34(4):439–59. | No intervention |
| Masterson-Algar P, Williams S, Burton CR, Arthur CA, Hoare Z, Morrison V, et al. Getting back to life after stroke: co-designing a peer-led coaching intervention to enable stroke survivors to rebuild a meaningful life after stroke. Disability & Rehabilitation. 2020;42(10):1359–72. | No physical activity |
| Marcum Z.A., Rosenberg D., Barnes D.E., Yaffe K., Larson E.B. Engaging Patients to Design the Systematic Multi-Domain Alzheimer’s Risk Reduction Trial (SMARRT) Intervention: Findings from a Web-Based Survey. Journal of Alzheimer’s Disease Reports. 2020;4(1):255–60. | No co-design |
| Marcucci M., Damanti S., Germini F., Apostolo J., Bobrowicz-Campos E., Gwyther H., et al. Interventions to prevent, delay or reverse frailty in older people: A journey towards clinical guidelines. BMC Medicine. 2019;17(1):193. | No intervention |
| Manikam L, Shah R, Reed K, Santini G, Lakhanpaul M. Using a co-production prioritization exercise involving South Asian children, young people and their families to identify health priorities requiring further research and public awareness. Health expectations : an international journal of public participation in health care and health policy. 2017;20(5):852–61. | Not older adults |
| Macintosh E, Kelly L, Smith SO. Care about Physical Activity (CAPA) improvement programme -improving older people’s quality of life through engagement in movement meaningful to them. International Journal of Integrated Care (IJIC). 2019;19(S1):1–2. | No intervention |
| Lyons KD, Newman R, Adachi-Mejia AM, Whipple J, Hegel MT. Content analysis of a participant-directed interventon to optimize activity engagement of older adult cancer survivors. OTJR: Occupation, Participation and Health. 2018;38(1):38–45. | No co-design |
| Loss J, Brew-Sam N, Metz B, Strobl H, Sauter A, Tittlbach S. Capacity Building in Community Stakeholder Groups for Increasing Physical Activity: Results of a Qualitative Study in Two German Communities. International journal of environmental research and public health. 2020;17(7). | Not older adults |
| Look MA, Kaholokula JK, Carvhalo A, Seto T, de Silva M. Developing a culturally based cardiac rehabilitation program: the HELA study. Progress in community health partnerships : research, education, and action. 2012;6(1):103–10. | Not older adults |
| Lock M, Post D, Dollman J, Parfitt G. Feasibility and Process Evaluation of a Need-Supportive Physical Activity Program in Aged Care Workers: The Activity for Well-Being Project. Frontiers in psychology. 2020;11(101550902):518413. | Not older adults |
| Lindsay-Smith G, Eime R, O’Sullivan G, Harvey J, van Uffelen JGZ. A mixed-methods case study exploring the impact of participation in community activity groups for older adults on physical activity, health and wellbeing. BMC geriatrics. 2019;19(1):243. | No co-design |
| Lindeman M, Smith R, Vrantsidis F, Gough J. Action research in aged care: a model for practice change and development. Geriaction. 2002;20(1):10–4. | Unable to retrieve full text |
| Lin S-C, Chen I-J, Yu W-R, Lee S-YD, Tsai T-I. Effect of a community-based participatory health literacy program on health behaviors and health empowerment among community-dwelling older adults: A quasi-experimental study. Geriatric nursing (New York, NY). 2019;40(5):494–501. | No co-design |
| Lewis M., Chondros P., Mihalopoulos C., Lee Y.Y., Gunn J.M., Harvey C., et al. The assertive cardiac care trial: A randomised controlled trial of a coproduced assertive cardiac care intervention to reduce absolute cardiovascular disease risk in people with severe mental illness in the primary care setting. Contemporary Clinical Trials. 2020;97:106143. | No physical activity |
| Levasseur M, Lefebvre H, Levert M-J, Lacasse-Bedard J, Desrosiers J, Therriault P-Y, et al. Personalized citizen assistance for social participation (APIC): A promising intervention for increasing mobility, accomplishment of social activities and frequency of leisure activities in older adults having disabilities. Archives of gerontology and geriatrics. 2016;64(8214379, 7ax):96–102. | No physical activity |
| Lehto P. Interactive CaringTV R supporting elderly living at home. The Australasian medical journal. 2013;6(8):425–9. | No physical activity |
| Krops LA, Folkertsma N, Hols DHJ, Geertzen JHB, Dijkstra PU, Dekker R. Target population’s requirements on a community-based intervention for stimulating physical activity in hard-to-reach physically disabled people: an interview study. Disability and rehabilitation. 2019;41(19):2272–9. | Not older adults |
| Krieger J, Rabkin J, Sharify D, Song L. High point walking for health: creating built and social environments that support walking in a public housing community. American Journal of Public Health. 2009;99(S3):S593-9. | Not older adults |
| Koelewijn-van Loon MS, van der Weijden T, Ronda G, van Steenkiste B, Winkens B, Elwyn G, et al. Improving lifestyle and risk perception through patient involvement in nurse-led cardiovascular risk management: a cluster-randomized controlled trial in primary care. Preventive medicine. 2010;50(1–2):35–44. | Not older adults |
| Knippenberg E, Timmermans A, Palmaers S, Spooren A. Use of a technology-based system to motivate older adults in performing physical activity: a feasibility study. BMC geriatrics. 2021;21(1):81. | No co-design |
| Kirk JW, Bodilsen AC, Tjornhoj-Thomsen T, Pedersen MM, Bandholm T, Husted RS, et al. A tailored strategy for designing the Walk-Copenhagen (WALK-Cph) intervention to increase mobility in hospitalised older medical patients: a protocol for the qualitative part of the WALK-Cph project. BMJ open. 2018;8(3):e020272. | No co-design |
| Kim S, Koniak-Griffin D, Flaskerud JH, Guarnero PA. The impact of lay health advisors on cardiovascular health promotion: using a community-based participatory approach. The Journal of cardiovascular nursing. 2004;19(3):192–9. | Not older adults |
| Kim KH, Linnan L, Campbell MK, Brooks C, Koenig HG, Wiesen C. The WORD (wholeness, oneness, righteousness, deliverance): a faith-based weight-loss program utilizing a community-based participatory research approach. Health education & behavior : the official publication of the Society for Public Health Education. 2008;35(5):634–50. | Not older adults |
| Kieffer EC, Willis SK, Odoms-Young AM, Guzman JR, Allen AJ, Two Feathers J, et al. Reducing disparities in diabetes among African-American and Latino residents of Detroit: the essential role of community planning focus groups. Ethnicity & disease. 2004;14(3 Suppl 1):S27-37. | Not older adults |
| Kandula N.R., Bernard V., Dave S., Ehrlich-Jones L., Counard C., Shah N., et al. The South Asian Healthy Lifestyle Intervention (SAHELI) trial: Protocol for a mixed-methods, hybrid effectiveness implementation trial for reducing cardiovascular risk in South Asians in the United States. Contemporary Clinical Trials. 2020;92:105995. | Not older adults |
| Jensen L.H., Eklund M.L., Husebo H.K., Nickelsen H., Zahl A.-G. Group-based voice and physical therapy for persons with Parkinson’s disease - an action research study. Journal of Parkinson’s Disease. 2019;9(1):137–8. | Improper study design |
| Jansson A.-S.B., Carlsson G. Physical activity on prescription at the time of stroke or transient ischemic attack diagnosis - from a patient perspective. Disability and rehabilitation. 2019:1–8. | No co-design |
| Irvine KN, Marselle MR, Melrose A, Warber SL. Group Outdoor Health Walks Using Activity Trackers: Measurement and Implementation Insight from a Mixed Methods Feasibility Study. International journal of environmental research and public health. 2020;17(7). | No co-design |
| Irvine K., Marselle M., Melrose A., Warber S. Group outdoor health walks using activity trackers: Measurement and implementation insight from a mixed-methods feasibility study. Global Advances in Health and Medicine. 2020;9:109. | No co-design |
| Ingrid B., Marsella A. Factors influencing exercise participation by clients in long-term care. Perspectives (Gerontological Nursing Association (Canada)). 2008;32(4):5–11. | No co-design |
| Hurley M., Pearson J., Walsh N., Carter D. Co-creating an online rehabilitation programme for people with chronic knee/hip pain. Annals of the Rheumatic Diseases. 2016;75(Supplement 2):895. | Abstract/supplement |
| Hunt J, Marshall AL, Jenkins D. Exploring the meaning of, the barriers to and potential strategies for promoting physical activity among urban Indigenous Australians. Health promotion journal of Australia : official journal of Australian Association of Health Promotion Professionals. 2008;19(2):102–8. | Not older adults |
| Hordam B, Boolsen MW. Patient involvement in own rehabilitation after early discharge. Scandinavian journal of caring sciences. 2017;31(4):859–66. | No co-design |
| Hooker SP, Cirill LA, Geraghty A. Evaluation of the walkable neighborhoods for seniors project in Sacramento County. Health promotion practice. 2009;10(3):402–10. | No co-design |
| Hooker S.P. California active aging project. Journal of Aging and Physical Activity. 2002;10(3):354–9. | No co-design |
| Hong YA, Goldberg D, Ory MG, Towne SDJ, Forjuoh SN, Kellstedt D, et al. Efficacy of a Mobile-Enabled Web App (iCanFit) in Promoting Physical Activity Among Older Cancer Survivors: A Pilot Study. JMIR cancer. 2015;1(1):e7. | No co-design |
| Hoglund M.W., Sadovsky R., Classie J. Engagement in life activities promotes healthy aging in men. Journal of Men’s Health. 2009;6(4):354–65. | Improper study design |
| Hoffmann KD, Walnoha A, Sloan J, Buddadhumaruk P, Huang H-H, Borrebach J, et al. Developing a Community-Based Tailored Exercise Program for People With Severe and Persistent Mental Illness. Progress in community health partnerships : research, education, and action. 2015;9(2):213–27. | Not older adults |
| Hoermann S., Hale L., Winser S.J., Regenbrecht H. Patient engagement and clinical feasibility of Augmented Reflection Technology for stroke rehabilitation. International Journal on Disability and Human Development. 2014;13(3):355–60. | Not older adults |
| Hirsch M. Community-based rehabilitation: From neurons to neighborhoods. Parkinsonism and Related Disorders. 2009;15(SUPPL 2):S2. | Improper study design |
| Henteleff A, Wall H. The HANS KAI Project: a community-based approach to improving health and well-being through peer support. Health promotion and chronic disease prevention in Canada : research, policy and practice. 2018;38(3):135–46. | Not older adults |
| Heisler M, Bouknight RR, Hayward RA, Smith DM, Kerr EA. The relative importance of physician communication, participatory decision making, and patient understanding in diabetes self-management. Journal of general internal medicine. 2002;17(4):243–52. | No intervention |
| Hedberg-Kristensson E., Dahlin Ivanoff S., Iwarsson S. Participation in the prescription process of mobility devices: Experiences among older patients. British Journal of Occupational Therapy. 2006;69(4):169–76. | No physical activity |
| Hebblethwaite S, Curley L. Exploring the Role of Community Recreation in Stroke Recovery Using Participatory Action Research and Photovoice. Therapeutic Recreation Journal. 2015;49(1):1–17. | No physical activity |
| Hartman SJ, Rosen RK. Breast cancer relatives’ physical activity intervention needs and preferences: qualitative results. BMC women’s health. 2017;17(1):36. | Not older adults |
| Hamilton C, McCluskey A, Hassett L, Killington M, Lovarini M. Patient and therapist experiences of using affordable feedback-based technology in rehabilitation: a qualitative study nested in a randomized controlled trial. Clinical rehabilitation. 2018;32(9):1258–70. | No co-design |
| Hallward L., Chemtob K., Lambert S.D., Duncan L.R. Prostate cancer survivors’ and caregivers’ experiences using behavior change techniques during a web-based self-management and physical activity program: A qualitative study. Journal of Clinical Medicine. 2020;9(10):1–16. | No co-design |
| Gupta SS, Aroni R, Teede H. Experiences and Perceptions of Physical Activity Among South Asian and Anglo-Australians With Type 2 Diabetes or Cardiovascular Disease. Qualitative Health Research. 2017;27(3):391–405. | No co-design |
| Gunn H, Endacott R, Haas B, Marsden J, Freeman J. Development of a balance, safe mobility and falls management programme for people with multiple sclerosis. Disability and rehabilitation. 2018;40(24):2857–66. | Not older adults |
| Guell C, Panter J, Griffin S, Ogilvie D. Towards co-designing active ageing strategies: A qualitative study to develop a meaningful physical activity typology for later life. Health expectations : an international journal of public participation in health care and health policy. 2018;21(5):919–26. | No intervention |
| Grant E., Hochman J., Summapund J., Zhong H., Guo Y., Estrin D., et al. Engagement and outcomes among older adults with mobile health (mHealth) cardiac rehabilitation: Pilot study. Journal of the American Geriatrics Society. 2018;66(Supplement 2):S284. | Abstract/supplement |
| Granbo R., Riiser E.S., Boulton E., Helbostad J.L., Saltvedt I., Oksengard A.R., et al. Development of exercise groups for persons with dementia. European Geriatric Medicine. 2019;10(Supplement 1):S71. | Abstract/supplement |
| Goodwin V.A., Hatton A. Ageing well: Developing innovation through codesign. Age and Ageing. 2019;48(Supplement 2). | Abstract/supplement |
| Goliath I., Lindqvist O., Tishelman C. Feasibility of different action-oriented techniques used with patients, family and staff in sweden to improve the end-of-life care environment in an action-research project. Palliative Medicine. 2018;32(1 Supplement 1):13. | Abstract/supplement |
| Glynn LG, Glynn F, Casey M, Wilkinson LG, Hayes PS, Heaney D, et al. Implementation of the SMART MOVE intervention in primary care: a qualitative study using normalisation process theory. BMC family practice. 2018;19(1):48. | Not older adults |
| Gitlin L.N., Chernett N.L., Harris L.F., Palmer D., Hopkins P., Dennis M.P. Harvest health: Translation of the chronic disease self-management program for older African Americans in a senior setting. Gerontologist. 2008;48(5):698–705. | No co-design |
| Garner-Purkis A, Alageel S, Burgess C, Gulliford M. A community-based, sport-led programme to increase physical activity in an area of deprivation: a qualitative case study. BMC public health. 2020;20(1):1018. | Not older adults |
| Fullagar S, Petris S, Sargent J, Allen S, Akhtar M, Ozakinci G. Action research with parkrun UK volunteer organizers to develop inclusive strategies. Health promotion international. 2020;35(5):1199–209. | Not older adults |
| Fried LP, Carlson MC, McGill S, Seeman T, Xue Q-L, Frick K, et al. Experience Corps: a dual trial to promote the health of older adults and children’s academic success. Contemporary clinical trials. 2013;36(1):1–13. | No physical activity |
| Fried L.P., Carlson M.C., Freedman M., Frick K.D., Glass T.A., Hill J., et al. A Social Model for Health Promotion for an Aging Population: Initial Evidence on the Experience Corps Model. Journal of Urban Health. 2004;81(1):64–78. | No co-design |
| Forthofer M, Burroughs-Girardi E, Stoisor-Olsson L, Wilcox S, Sharpe PA, Pekuri LM. Use of formative research and social network theory to develop a group walking intervention: Sumter County on the Move!. Evaluation and program planning. 2016;58(7801727, eob):28–34. | Not older adults |
| Farahani LA, Parvizy S, Asadi-Lari M, Mohammadi E, Azghadi BH, Taghizadeh Z. Study protocol for promoting physical activity among women based on the MAPP process. Eastern Mediterranean health journal = La revue de sante de la Mediterranee orientale = al-Majallah al-sihhiyah li-sharq al-mutawassit. 2019;24(11):1074–81. | Not older adults |
| Fang ML, Woolrych R, Sixsmith J, Canham S, Battersby L, Sixsmith A. Place-making with older persons: Establishing sense-of-place through participatory community mapping workshops. Social science & medicine (1982). 2016;168(ut9, 8303205):223–9. | No physical activity |
| Fabbri L., Mosca I.E., Gerli F., Martini L., Pancani S., Lucidi G., et al. The Games for Older Adults Active Life (GOAL) project for people with mild cognitive impairment and vascular cognitive impairment: A study protocol for a randomized controlled trial. Frontiers in Neurology. 2019;10(JAN):1040. | No co-design |
| Esfehani A.J., Dashti S. Barriers to exercise participation among dialysis patients. Nephrology Dialysis Transplantation. 2012;27(10):3964. | No co-design |
| Ellard DR, Thorogood M, Underwood M, Seale C, Taylor SJC. Whole home exercise intervention for depression in older care home residents (the OPERA study): a process evaluation. BMC medicine. 2014;12(101190723):1. | No co-design |
| Duvall J. A comparison of engagement strategies for encouraging outdoor walking. Journal of physical activity & health. 2012;9(1):62–70. | Not older adults |
| Downey SM, Wages J, Jackson SF, Estabrooks PA. Adoption decisions and implementation of a community-based physical activity program: a mixed methods study. Health promotion practice. 2012;13(2):175–82. | Not older adults |
| Dodd-Reynolds CJ, Nevens L, Oliver EJ, Finch T, Lake AA, Hanson CL. Prototyping for public health in a local context: a streamlined evaluation of a community-based weight management programme (Momenta), Northumberland, UK. BMJ open. 2019;9(10):e029718. | No co-design |
| Dobbins S, Hubbard E, Leutwyler H. ‘Looking Forward’: a qualitative evaluation of a physical activity program for middle-aged and older adults with serious mental illness. International psychogeriatrics. 2020;32(12):1449–56. | Not older adults |
| Dixon-Ibarra A, Driver S, Vanderbom K, Humphries K. Understanding physical activity in the group home setting: a qualitative inquiry. Disability & Rehabilitation. 2017;39(7):653–62. | Not older adults |
| Dacey M.L., Newcomer A.R. A client-centered counseling approach for motivating older adults toward physical activity. Topics in Geriatric Rehabilitation. 2005;21(3):194–205. | Improper study design |
| D. Almevall A, Zingmark K, Nordmark S, Forslund A-S, Niklasson J. Accepting the inevitable: A mixed method approach with assessment and perceptions of well-being in very old persons within the northern Sweden Silver-MONICA study. Archives of Gerontology & Geriatrics. 2021;92:N.PAG-N.PAG. | No intervention |
| Cress M.E., Buchner D.M., Prohaska T., Rimmer J., Brown M., Macera C., et al. Best practices for physical activity programs and behavior counseling in older adult populations. Journal of Aging and Physical Activity. 2005;13(1):61–74. | No co-design |
| Corrado AM, Benjamin-Thomas TE, McGrath C, Hand C, Laliberte Rudman D. Participatory Action Research With Older Adults: A Critical Interpretive Synthesis. The Gerontologist. 2020;60(5):e413–27. | Improper study design |
| Collins T.C., Kroll T.L., Krueger P.N., Willson P., Ashton C.M., Sharf B.F. A qualitative approach to developing a patient-derived intervention to increase exercise in peripheral arterial disease. Journal of Cardiopulmonary Rehabilitation. 2006;26(2):92–100. | No intervention |
| Clift E., Roberts H., Sayer A., Rogers A. Who uses exercise in later life? European Geriatric Medicine. 2017;8(Supplement 1):S209. | Abstract/supplement |
| Clarke A.L., MacKinnon H.J., Yates T., Smith A.C. The ‘person-based approach’ to developing a structured group education programme to increase physical activity in CKD: The pact-project. Nephrology Dialysis Transplantation. 2016;31(SUPPL. 1). | Abstract/supplement |
| Clark AM, Barbour RS, White M, MacIntyre PD. Promoting participation in cardiac rehabilitation: patient choices and experiences. Journal of advanced nursing. 2004;47(1):5–14. | No co-design |
| Clark A.M., Mundy C., Catto S., MacIntyre P.D. Participation in community-based exercise maintenance programs after completion of hospital-based cardiac rehabilitation: A mixed-method study. Journal of Cardiopulmonary Rehabilitation and Prevention. 2011;31(1):42–6. | No intervention |
| Ciro CA, Smith P. Improving Personal Characterization of Meaningful Activity in Adults with Chronic Conditions Living in a Low-Income Housing Community. International journal of environmental research and public health. 2015;12(9):11379–95. | No physical activity |
| Cinderby S., Cambridge H., Attuyer K., Bevan M., Croucher K., Gilroy R., et al. Co-designing Urban Living Solutions to Improve Older People’s Mobility and Well-Being. Journal of urban health : bulletin of the New York Academy of Medicine. 2018;95(3):409–22. | No physical activity |
| Chisholm M., Greenland R., Shilton T., Salmon J. Heart foundation’s blueprint for an active Australia 2014-2017: Power through partnership-Collective action toward increasing Australia’s physical activity levels. Journal of Science and Medicine in Sport. 2014;18(SUPPL. 1):e26. | Not older adults |
| Cheadle A, Egger R, LoGerfo JP, Schwartz S, Harris JR. Promoting sustainable community change in support of older adult physical activity: evaluation findings from the Southeast Seattle Senior Physical Activity Network (SESPAN). Journal of urban health : bulletin of the New York Academy of Medicine. 2010;87(1):67–75. | Not older adults |
| Che Hasan MK, Stanmore E, Todd C. Perspectives of ESCAPE-Pain Programme for Older People With Knee Osteoarthritis in the Community Setting. Frontiers in public health. 2020;8(101616579):612413. | No co-design |
| Celinder D, Peoples H. Stroke patients’ experiences with Wii Sports® during inpatient rehabilitation. Scandinavian Journal of Occupational Therapy. 2012;19(5):457–63. | No co-design |
| Cash MF, Ulanowski E, Danzl M. Development of a community-based golf and exercise program for people with Parkinson’s disease. Complementary therapies in clinical practice. 2018;33(101225531):149–55. | No co-design |
| Carron T, Bridevaux P-O, Lorvall K, Parmentier R, Moix J-B, Beytrison V, et al. Feasibility, acceptability and effectiveness of integrated care for COPD patients: a mixed methods evaluation of a pilot community-based programme. Swiss medical weekly. 2017;147(d10, 100970884):w14567. | No co-design |
| Carroll J., Hopper L. Engaging older adults in co-creating a virtual coaching assistant (CAPTAIN) to support independent living at home. Age and Ageing. 2019;48(Supplement 3). | Abstract/supplement |
| Canella C., Mikolasek M., Rostock M., Beyer J., Guckenberger M., Jenewein J., et al. Developing an Integrative Treatment Program for Cancer-Related Fatigue Using Stakeholder Engagement - A Qualitative Study. Integrative Cancer Therapies. 2018;17(3):762–73. | Not older adults |
| Byrne L, Ogden K, Lee S, Ahuja K, Watson G, Bauman A, et al. Mixed-method evaluation of a community-wide physical activity program in Launceston, Australia. Health Promotion Journal of Australia. 2019;30:104–15. | Not older adults |
| Byrne L, Ogden K, Lee S, Ahuja K, Watson G, Bauman A, et al. Mixed-method evaluation of a community-wide physical activity program in Launceston, Australia. Health promotion journal of Australia : official journal of Australian Association of Health Promotion Professionals. 2019;30 Suppl 1(9710936):104–15. | Not older adults |
| Buttery A.K., Martin F.C. Knowledge, attitudes and intentions about participation in physical activity of older post-acute hospital inpatients. Physiotherapy. 2009;95(3):192–8. | No co-design |
| Burford S, Park S, Dawda P, Burns J. Participatory research design in mobile health: Tablet devices for diabetes self-management. Communication & medicine. 2015;12(2–3):145–56. | Not older adults |
| Buckley BJ, Thijssen DH, Murphy RC, Graves LE, Whyte G, Gillison F, et al. Preliminary effects and acceptability of a co-produced physical activity referral intervention. Health Education Journal. 2019;78(8):869–84. | Not older adults |
| Brown L., Campbell K., Kyle R. Co-designing with older adults living with cancer: Exploring the strengths and limitations. Psycho-Oncology. 2020;29(Supplement 2):27. | Abstract/supplement |
| Brown L., Campbell K., Kyle R. Co-designing a behaviour change intervention for older adults living with cancer: Initial insights. Psycho-Oncology. 2019;28(Supplement 2):21. | Abstract/supplement |
| Browne J, Medenblik A, Pebole M, Gregg JJ, Hall KS. Qualitative Analysis of a Supervised Exercise Program for Older Veterans With PTSD. The American journal of geriatric psychiatry : official journal of the American Association for Geriatric Psychiatry. 2020;(cm3, 9309609). | No co-design |
| Bridges J. Action research in rehabilitation care for older people. Nursing Times. 2001;97(5):37–8. | No physical activity |
| Bradbury K., Steele M., Corbett T., Geraghty A.W.A., Krusche A., Heber E., et al. Developing a digital intervention for cancer survivors: an evidence-, theory- and person-based approach. npj Digital Medicine. 2019;2(1):85. | Improper study design |
| Boulton ER, Horne M, Todd C. Multiple influences on participating in physical activity in older age: Developing a social ecological approach. Health expectations : an international journal of public participation in health care and health policy. 2018;21(1):239–48. | No intervention |
| Binns E., Kerse N., Peri K., Cheung G., Taylor D. Combining cognitive stimulation therapy and fall prevention exercise (CogEx) in older adults with mild to moderate dementia: A feasibility randomised controlled trial. Pilot and Feasibility Studies. 2020;6(1):108. | No co-design |
| Bharmal N., Mittman B.S., Shah R., Sarkisian C. Cultural tailoring of stroke risk factor reduction and walking promotion community intervention for South Asian older adult immigrants. Journal of General Internal Medicine. 2014;29(SUPPL. 1):S60–1. | Abstract/supplement |
| Bertelsen P, Kanstrup Am, Madsen J. Steps Toward Technology Design to Beat Health Inequality -- Participatory Design Walks in a Neighbourhood with High Health Risks. Studies in Health Technology & Informatics. 2017;233:158–72. | Not older adults |
| Berends L, Halliday R. Capacity building and social marketing promotes healthy lifestyle behaviour in an Australian Aboriginal community. The Australian journal of rural health. 2018;(9305903, c0i). | Not older adults |
| Benzo R, Vickers K, Ernst D, Tucker S, McEvoy C, Lorig K. Development and feasibility of a self-management intervention for chronic obstructive pulmonary disease delivered with motivational interviewing strategies. Journal of cardiopulmonary rehabilitation and prevention. 2013;33(2):113–23. | No co-design |
| Bennett S., Ballinger C. Considering older people’s views is important in gaining and maintaining participation in falls prevention interventions: Commentary. Australian Occupational Therapy Journal. 2006;53(3):241–3. | Improper study design |
| Bennett J.A., Winters-Stone K. Motivating older adults to exercise: What works? Age and Ageing. 2011;40(2):148–9. | No intervention |
| Benedetti TRB, Rech CR, Konrad LM, Almeida FA, Brito FA, Chodzko-Zajko W, et al. Re-thinking Physical Activity Programs for Older Brazilians and the Role of Public Health Centers: A Randomized Controlled Trial Using the RE-AIM Model. Frontiers in public health. 2020;8(101616579):48. | No-codesign |
| Beekman E, Braun SM, Ummels D, van Vijven K, Moser A, Beurskens AJ. Validity, reliability and feasibility of commercially available activity trackers in physical therapy for people with a chronic disease: a study protocol of a mixed methods research. Pilot and feasibility studies. 2017;3(101676536):64. | Not older adults |
| Baretta D, Perski O, Steca P. Exploring Users’ Experiences of the Uptake and Adoption of Physical Activity Apps: Longitudinal Qualitative Study. JMIR mHealth and uHealth. 2019;7(2):e11636. | Not older adults |
| Baccellieri A., Gruss V., Hasnain M., Zarrieneh A., Koronkowski M. Connecting the dots: Participatory health education and empowerment program with older adults. Journal of the American Geriatrics Society. 2017;65(Supplement 1):S123. | Abstract/supplement |
| Attwood S, Morton KL, Mitchell J, Van Emmenis M, Sutton S, VBI Programme Team. Reasons for non-participation in a primary care-based physical activity trial: a qualitative study. BMJ open. 2016;6(5):e011577. | Not older adults |
| Arnold A., Holmes E., Rosenthal M. Building a patient-centered weight management program: A mixed methods project to obtain patients’ information needs and ideas for program structure. Pharmacy. 2019;7(4):165. | Not older adults |
| Ardo J.C., Lee J.-A., Hildebrand J., Guijarro D., Ghasemzadeh H.G., Evangelista L.S. A Co-design approach to validate a text message bank for use with older adults at risk of cardiovascular disease. Circulation. 2019;139(Supplement 1). | Abstract/supplement |
| Aramaki AL, Sampaio RF, Cavalcanti A, Dutra FCMSE. Use of client-centered virtual reality in rehabilitation after stroke: a feasibility study. Arquivos de neuro-psiquiatria. 2019;77(9):622–31. | Not older adults |
| Apps LD, Mitchell KE, Harrison SL, Sewell L, Williams JE, Young HM, et al. The development and pilot testing of the self-management programme of activity, coping and education for chronic obstructive pulmonary disease (SPACE for COPD). International journal of chronic obstructive pulmonary disease. 2013;8(101273481):317–27. | No physical activity |
| Anonymous. New four-pronged approach to wellness combines an eclectic mix of strategies. Disease management advisor. 2006;12(9):105–97. | No co-design |
| Anderson LA, Slonim A, Yen IH, Jones DL, Allen P, Hunter RH, et al. Developing a framework and priorities to promote mobility among older adults. Health education & behavior : the official publication of the Society for Public Health Education. 2014;41(1 Suppl):10S-8S. | No co-design |
| Ali A., Church G., Bec R., Langley J. Enhancing uptake of an exercise programme after stroke or transient ischaemic attack (TIA)-a co-production event. International Journal of Stroke. 2018;13(3 Supplement 1):29. | Abstract/supplement |
| Akintobi TH, Lockamy E, Goodin L, Hernandez ND, Slocumb T, Blumenthal D, et al. Processes and Outcomes of a Community-Based Participatory Research-Driven Health Needs Assessment: A Tool for Moving Health Disparity Reporting to Evidence-Based Action. Progress in community health partnerships : research, education, and action. 2018;12(1S):139–47. | No intervention; |
| Adu MD, Malabu UH, Malau-Aduli AEO, Malau-Aduli BS. Users’ preferences and design recommendations to promote engagements with mobile apps for diabetes self-management: Multi-national perspectives. PloS one. 2018;13(12):e0208942. | No intervention |
| Adsett J., Hickey A., Nagle A., Mudge A. Implementing a community-based model of exercise training following cardiac, pulmonary, and heart failure rehabilitation. Journal of Cardiopulmonary Rehabilitation and Prevention. 2013;33(4):239–43. | No co-design |
| Adams SA, Heiney SP, Brandt HM, Wirth MD, Khan S, Johnson H, et al. A comparison of a centralized versus de-centralized recruitment schema in two community-based participatory research studies for cancer prevention. Journal of community health. 2015;40(2):251–9. | Not older adults |
| Aartolahti E, Hartikainen S, Lonnroos E, Hakkinen A. Health and physical function predicting strength and balance training adoption: a community-based study among individuals aged 75 and older. Journal of aging and physical activity. 2014;22(4):543–9. | No co-design |
