# Supplementary material for: The use of co-design in developing physical activity interventions for older adults: a scoping review
Source: BMC Geriatr. 2022 Aug 8;22:647. doi: 10.1186/s12877-022-03345-4 (PMC9358386; doi:10.1186/s12877-022-03345-4)
Supplement: Supplementary file 6 — Additional file 6. Operational definitions of co-design terminology extracted. [file 12877_2022_3345_MOESM6_ESM.docx]

Additional File 6: Summary of Co-Design Terminology and Operational Definitions

| Terminology | Operational definitions |
| --- | --- |
| Co-design (1–4) | - “Co-design as a process of information system development aims at transferring the focus from the technology towards the user. One stated aim of using co-design is to move the user to the center of an information system development project.” (1,5) - “A technique in which potential end users in partnership with other stakeholders and researchers work together in all stages of product development, from needs assessment, content definition, prototyping, testing and dissemination [*allowing*] for conceptual re-development and strategies based on the socio-cultural needs of different groups.” (2,6) - “Stakeholder consultation as a mandatory step to create a context specific, fit-for-purpose, user-centered approach. Co-design goes it is a person-centered ethos sought to empower patients to tailor an intervention to suit their own contexts and partner in creating an intervention that would be appropriate and responsive to their needs […]This involved iterative processes of user engagement where intervention codesign was viewed as a partnership.” (4,7,8) |
| Co-creation (2,9–16) | - “[…] Interventions should be tailored to an individual’s context and circumstance to improve adherence. Co-creation has been recommended as an approach to develop such interventions by working with older adults to ensure developed solutions are congruent with their daily lives.”(9) - “Co-creation is known as a creative and collaborative activity. People with shared goals, but different skills and knowledge, collaborate together through an interdisciplinary process. Often it can be observed that in network innovations values are created through co-creation for the end users via direct or indirect interactions be-tween many different partners at the network level. […]By synchronizing the different expectations of the stakeholders and focusing on the end user insights, shared values and joint propositions for the intended target users can be created.” (10) - “The process of co-creation, which may be defined as collaboration between different groups of stakeholders to develop a mutually beneficial outcome, has been shown to increase individual satisfaction, improve awareness of individual health behaviours, improve the effectiveness of programmes and enhance the quality of service provision.” (11) - “A promising approach […] is to involve the end-users early and frequently during the develop-mental process through a user-centred design (UCD) approach. This is an iterative, cyclical process during which design and evaluation phases are alternated. In this way, systems and services can be developed step by step, so that changes in technology and work process can evolve together, and unforeseen challenges can be easily anticipated in future development steps.” (12) - “The terminology co-creation […] *corresponds* to a process where the end-user is engaged in the development or design of a product. […] Participatory design *deals* with the mutual learning and mixed roles of designer and user when working together in a creative way. We define our co-creation process as an interaction with participants during sessions where prototypes were elaborated on. Suggestions from each session led to developments of the application’s user interface and instructions, which were further processed at the subsequent session.”(14) - “Emergent from the participatory design paradigm is a process called co-creation, which […] shifts the design process from the traditional “top-down” health model to an inductive paradigm of shared leadership allowing end-users to take control over the content of the activities, and be involved in their health management and decision-making relevant to their own health.” (15) |
| Action Research (16–20) | - “consultation and reflection shapes future action […] *while* employing a continuous cycle of evaluation and revision, emphasizing the democratic and participative aspects of the action research process, ensuring that participants decided on what was most important for their community, building interventions in partnership with the […] researcher and gaining ownership of the project.” (19) |
| Participatory design (14,21–23) | - “The terminology […] participatory design *corresponds* to a process where the end-user is engaged in the development or design of a product. […] Participatory design *deals* with the mutual learning and mixed roles of designer and user when working together in a creative way.”(14) - “In the tradition of participatory […] *design* researchers and users collaborate in the design process, where users serve as experts of their own experience and design researchers as experts in translating user experiences to viable design opportunities.”(21) - “Researchers have drawn upon a variety of user-centred and participatory design methodologies to facilitate collaboration and engagement with older adults. These include creative activities, such as sketching, paper prototyping, and model and mock-up making; exploratory activities, such as card sorting, storyboarding and diary keeping; and playful activities, such as design gaming, scenario-based role-playing, and future thinking and envisioning.” (22) |
| Community Based Participatory Research /Participatory research (24–27) | - “host sites, program participants, and researchers could collaboratively develop a program that met user needs” (25) - “We adopted a community-based participatory research technique known as photo-elicitation inviting participants to take photographs of salient and personally meaningful features in their lives. Visual images gathered from photo-elicitation were explored in detail through in-depth interviews in the second phase.” (26) - “it actively involves the community as well as communal stakeholders” (24) |
| Engagement (9,11,13,28) | - NR |
| User centered design (12,23,29) | - “This is an iterative, cyclical process during which design and evaluation phases are alternated. In this way, systems and services can be developed step by step, so that changes in technology and work process can evolve together, and unforeseen challenges can be easily anticipated in future development steps.” (12) - “The goal of user-centered design (UCD) is to understand the users’ perspective and to use that knowledge to shape more effective solutions. The UCD approach provides insight into users’ needs and requirements and thereby improves the design of the developed services. However, involving users in the development process does not guarantee that feedback from different subgroups of users will shape the development in ways that will make the solutions more useful for the entire target user population.” (29) - “User-centered design (UCD) is a process (not restricted to interfaces or technologies) in which the needs, wants, and limitations of end users of a product, service, or process are given extensive attention at each stage of the design process. UCD can be characterized as a multistage problem-solving process that not only requires designers to analyze and foresee how users are likely to use a product but also to test the validity of their assumptions with regard to user behavior in real world tests with actual users.”(23,30) |
| Participatory action research (PAR) (31,32) | - “PAR is a design that brings together researchers and stakeholders (e.g., residents, civil groups, professionals, company, and government) in a collaborative effort to address issues in specific systems. It is a collaborative, cyclical (analogous to “plan-do-check-act”) and reflective inquiry design that focuses on problem solving and understanding the effect of an intervention as part of the research process.” (31) - “Participatory action research (PAR) is a methodology that enables researchers [to] work in partnership with communities in a manner that leads to action for change [..] researchers and participants can thus co-identify important issues, promote open dialogue and community participation, and bridge gaps in knowledge and practice between researchers and communities.” (32) |
| Integrated Knowledge Translation (33) | - “Integrated knowledge translation is defined as a collaboration between researchers and people who are influenced by the research, which includes a range of decision-makers. This approach ensures that stakeholder voices are heard and enables development of programs that can be sustained outside a research framework.” (33) |
| Qualitative methodology (34) | - “*An* approach to examine what older adults would like from a PA intervention, and how PA programmes targeting older adults should be delivered to be acceptable for them” (34) |
| User-involvement (35) | - “Involvement of potential users at all stages of the development of the device.” (35) |
| Co-learning (20) | - “Co-learning is hereby defined as interactive processes that can result in the involved individuals gaining new information and perspectives, which when accumulated can increase their knowledge and change their way of thinking.” (20) |
| Stakeholder involvement (36) | - “Stakeholders are “individuals or groups who are responsible for or affected by health- and healthcare-related decisions that can be informed by research evidence”[…] involvement should occur throughout the research process (i.e., in the preparation, execution and translation phases).”(36) |

References:

1. Da Silva Júnior JLA, Biduski D, Bellei EA, Becker OHC, Daroit L, Pasqualotti A, et al. A Bowling Exergame to Improve Functional Capacity in Older Adults: Co-Design, Development, and Testing to Compare the Progress of Playing Alone Versus Playing With Peers. JMIR Serious Games [Internet]. 2021 Jan 29 [cited 2021 Jun 6];9(1). Available from: https://www.ncbi.nlm.nih.gov/pmc/articles/PMC7880815/

2. Castro PC, Romano LB, Frohlich D, Lorenzi LJ, Campos LB, Paixão A, et al. Tailoring digital apps to support active ageing in a low income community. PLOS ONE. 2020 Dec 10;15(12):e0242192.

3. Pearson J, Walsh N, Carter D, Koskela S, Hurley M. Developing a Web-Based Version of An Exercise-Based Rehabilitation Program for People With Chronic Knee and Hip Pain: A Mixed Methods Study. JMIR Res Protoc [Internet]. 2016 May 19 [cited 2021 Jun 6];5(2). Available from: https://www.ncbi.nlm.nih.gov/pmc/articles/PMC4891573/

4. Walsh DMJ, Moran K, Cornelissen V, Buys R, Claes J, Zampognaro P, et al. The development and codesign of the PATHway intervention: a theory-driven eHealth platform for the self-management of cardiovascular disease. Transl Behav Med. 2019 Jan 1;9(1):76–98.

5. Askenäs L, Aidemark J, Jaarsma T, Sttrömberg A, Klompstra L. CO-DESIGN TO SELF-ORGANIZING EXERGAMING - A STUDY OF STIMULATING PHYSICAL ACTIVITY FOR ELDERLY PEOPLE WITH A CHRONIC HEALTH CONDITION. In 2019. p. 35–41.

6. Boyd H, McKernon S, Mullin B, Old A. Improving healthcare through the use of co-design. N Z Med J. 2012 Jun 29;125(1357):76–87.

7. Cornwall A, Jewkes R. What is participatory research? Soc Sci Med. 1995 Dec;41(12):1667–76.

8. Jagosh J, Macaulay AC, Pluye P, Salsberg J, Bush PL, Henderson J, et al. Uncovering the benefits of participatory research: implications of a realist review for health research and practice. Milbank Q. 2012 Jun;90(2):311–46.

9. Leask CF, Sandlund M, Skelton DA, Chastin SF. Co-creating a tailored public health intervention to reduce older adults’ sedentary behaviour. Health Education Journal. 2017 Aug 1;76(5):595–608.

10. Lu Y, Valk C, Steenbakkers J, Bekker T, Visser T, Proctor G, et al. Can technology adoption for older adults be co-created? Gerontechnology. 2017 Nov 7;16:151–9.

11. Leask CF, Colledge N, Laventure RME, McCann DA, Skelton DA. Co-Creating Recommendations to Redesign and Promote Strength and Balance Service Provision. Int J Environ Res Public Health [Internet]. 2019 Sep [cited 2021 Jun 6];16(17). Available from: https://www.ncbi.nlm.nih.gov/pmc/articles/PMC6747106/

12. Timmerman JG, Tönis TM, Dekker-van Weering MGH, Stuiver MM, Wouters MWJM, van Harten WH, et al. Co-creation of an ICT-supported cancer rehabilitation application for resected lung cancer survivors: design and evaluation. BMC Health Services Research. 2016 Apr 27;16(1):155.

13. Tabak M, de Vette F, van Dijk H, Vollenbroek-Hutten M. A Game-Based, Physical Activity Coaching Application for Older Adults: Design Approach and User Experience in Daily Life. Games Health J. 2020 Jun;9(3):215–26.

14. Mansson L, Wiklund M, Öhberg F, Danielsson K, Sandlund M. Co-Creation with Older Adults to Improve User-Experience of a Smartphone Self-Test Application to Assess Balance Function. Int J Environ Res Public Health [Internet]. 2020 Jun [cited 2021 Jun 6];17(11). Available from: https://www.ncbi.nlm.nih.gov/pmc/articles/PMC7312460/

15. Giné-Garriga M, Dall PM, Sandlund M, Jerez-Roig J, Chastin SFM, Skelton DA. A Pilot Randomised Clinical Trial of a Novel Approach to Reduce Sedentary Behaviour in Care Home Residents: Feasibility and Preliminary Effects of the GET READY Study. Int J Environ Res Public Health [Internet]. 2020 Apr [cited 2021 Jun 6];17(8). Available from: https://www.ncbi.nlm.nih.gov/pmc/articles/PMC7215704/

16. Giné-Garriga M, Sandlund M, Dall PM, Chastin SFM, Pérez S, Skelton DA. A Novel Approach to Reduce Sedentary Behaviour in Care Home Residents: The GET READY Study Utilising Service-Learning and Co-Creation. Int J Environ Res Public Health [Internet]. 2019 Feb [cited 2021 Jun 6];16(3). Available from: https://www.ncbi.nlm.nih.gov/pmc/articles/PMC6388363/

17. Kittipimpanon K, Amnatsatsue K, Kerdmongkol P, Maruo SJ, Nityasuddhi D. Development and Evaluation of a Community-based Fall Prevention Program for Elderly Thais Development and Evaluation of a Community-based Fall Prevention Program for Elderly Thais. 2012.

18. Jitramontree N, Chatchaisucha S, Thaweeboon T, Kutintara B, Intanasak S. Action Research Development of a Fall Prevention Program for Thai Community-dwelling Older Persons. Pacific Rim International Journal of Nursing Research. 2015 Feb 10;19(1):69–79.

19. Davies J, Lester C, O’Neill M, Williams G. Sustainable participation in regular exercise amongst older people: Developing an action research approach. Health Education Journal. 2008 Mar 1;67(1):45–55.

20. Åberg AC, Halvorsen K, From I, Bruhn ÅB, Oestreicher L, Melander-Wikman A. A Study Protocol for Applying User Participation and Co-Learning-Lessons Learned from the eBalance Project. Int J Environ Res Public Health. 2017 May 10;14(5).

21. Verhoeven F, Cremers A, Schoone M, Dijk J van. Mobiles for mobility: Participatory design of a ‘Happy walker’ that stimulates mobility among older people. GERONTOLOGY. 2016;15(1):32–44.

22. Swallow D, Petrie H, Power C, Lewis A, Edwards ADN. Involving Older Adults in the Technology Design Process: A Case Study on Mobility and Wellbeing in the Built Environment. Stud Health Technol Inform. 2016;229:615–23.

23. Brox E, Konstantinidis ST, Evertsen G. User-Centered Design of Serious Games for Older Adults Following 3 Years of Experience With Exergames for Seniors: A Study Design. JMIR Serious Games [Internet]. 2017 Jan 11 [cited 2021 Jun 5];5(1). Available from: https://www.ncbi.nlm.nih.gov/pmc/articles/PMC5266825/

24. Bammann K, Recke C, Albrecht BM, Stalling I, Doerwald F. Promoting Physical Activity Among Older Adults Using Community-Based Participatory Research With an Adapted PRECEDE-PROCEED Model Approach: The AEQUIPA/OUTDOOR ACTIVE Project. Am J Health Promot. 2021 Mar 1;35(3):409–20.

25. Parker SJ, Chen EK, Pillemer K, Filiberto D, Laureano E, Piper J, et al. Participatory Adaptation of an Evidence-Based, Arthritis Self-Management Program: Making Changes to Improve Program Fit. Fam Community Health. 2012;35(3):236–45.

26. Balbale S, Schwingel A, Chodzko-Zajko W, Huhman M. Visual and Participatory Research Methods for the Development of Health Messages for Underserved Populations. Health communication. 2013 Oct 30;29.

27. Bammann K, Drell C, Lübs LL, Stalling I. Cluster-randomised trial on participatory community-based outdoor physical activity promotion programs in adults aged 65–75 years in Germany: protocol of the OUTDOOR ACTIVE intervention trial. BMC Public Health [Internet]. 2018 Oct 23 [cited 2021 Jun 6];18. Available from: https://www.ncbi.nlm.nih.gov/pmc/articles/PMC6199784/

28. Browne J, Medenblik A, Pebole M, Gregg JJ, Hall KS. Qualitative Analysis of a Supervised Exercise Program for Older Veterans With PTSD. The American Journal of Geriatric Psychiatry. 2021 Jun 1;29(6):565–72.

29. Revenäs Å, Johansson A-C, Ehn M. Integrating Key User Characteristics in User-Centered Design of Digital Support Systems for Seniors’ Physical Activity Interventions to Prevent Falls: Protocol for a Usability Study. JMIR Res Protoc [Internet]. 2020 Dec 21 [cited 2021 Jun 6];9(12). Available from: https://www.ncbi.nlm.nih.gov/pmc/articles/PMC7781794/

30. Sebe N. Human-centered Computing. In: Nakashima H, Aghajan H, Augusto JC, editors. Handbook of Ambient Intelligence and Smart Environments [Internet]. Boston, MA: Springer US; 2010 [cited 2021 Jun 6]. p. 349–70. Available from: https://doi.org/10.1007/978-0-387-93808-0_13

31. Seino S, Kitamura A, Tomine Y, Tanaka I, Nishi M, Nonaka K, et al. A Community-Wide Intervention Trial for Preventing and Reducing Frailty Among Older Adults Living in Metropolitan Areas: Design and Baseline Survey for a Study Integrating Participatory Action Research With a Cluster Trial. J Epidemiol. 2019 Feb 5;29(2):73–81.

32. Frigault JS, Giles AR. Culturally Safe Falls Prevention Program for Inuvialuit Elders in Inuvik, Northwest Territories, Canada: Considerations for Development and Implementation. Canadian Journal on Aging / La Revue canadienne du vieillissement. 2020 Jun;39(2):190–205.

33. Bird M-L, Mortenson BW, Chu F, Acerra N, Bagnall E, Wright A, et al. Building a Bridge to the Community: An Integrated Knowledge Translation Approach to Improving Participation in Community-Based Exercise for People After Stroke. Phys Ther. 2019 Mar;99(3):286–96.

34. Arnautovska U, O’callaghan F, Hamilton K. Behaviour change techniques to facilitate physical activity in older adults: what and how. Ageing & Society. 2018 Dec;38(12):2590–616.

35. Lacey G, MacNamara S. User involvement in the design and evaluation of a smart mobility aid. J Rehabil Res Dev. 2000 Dec;37(6):709–23.

36. Brach JS, Perera S, Gilmore S, VanSwearingen JM, Brodine D, Wert D, et al. Stakeholder involvement in the design of a patient-centered comparative effectiveness trial of the “On the Move” group exercise program in community-dwelling older adults. Contemp Clin Trials. 2016 Sep;50:135–42.
